# Nitrate/nitrite-driven methane oxidation enhanced N_2_O production in marine cold seeps

**DOI:** 10.1128/mbio.01446-26

**Published:** 2026-07-31

**Authors:** Weikang Sui, Longhui Deng, Bing-Zheng Wu, Guang-Chao Zhuang, Zian Tong, Ningyuan Lu, Danyue Huang, Zheng Xiong, Peng Guo, Xianbiao Lin, Yanwei Wang, Liang Dong, Fengping Wang

**Affiliations:** 1 State Key Laboratory of Submarine Geoscience, Key Laboratory of Polar Ecosystem and Climate Change, Ministry of Education, Shanghai Key Laboratory of Polar Life and Environment Sciences and School of Oceanography, Shanghai Jiao Tong University 12474, Shanghai, China; 2 Frontiers Science Center for Deep Ocean Multispheres and Earth System and Key Laboratory of Marine Chemistry Theory and Technology, Ministry of Education, Ocean University of China, Qingdao, China; Georgia Institute of Technology, Atlanta, Georgia, USA

**Keywords:** cold seep, stage-dependent dynamics, nitrate/nitrite-coupled methane oxidation, methane-oxidizing bacteria, nitrous oxide

## Abstract

Methane oxidation coupled to sulfate reduction is the dominant process restricting methane release from cold seeps. Methane oxidation can also be coupled to nitrate/nitrite reduction, thereby linking the cycles of CH_4_, CO_2_, and N_2_O. However, how this coupling shifts during seep development and its impact on N_2_O production remains poorly understood. Here, we integrated geochemical profiling, ^14^C-based labeling, and omics-based analyses to quantify the process rates, identify the key microbial mediators, and assess the net N_2_O production across the developmental stages of cold seeps in the South China Sea. In an early-stage, polychaete-dominated seep, the depth-integrated, potential nitrate/nitrite-coupled methane oxidation rate reached 21.9 ± 3.0 mmol C/m^2^/day, accounting for 36%–73% of the total methane oxidation. By contrast, in a typical epifauna-prevalent mature seep, this contribution diminished to ~1% (0.62 ± 0.16 mmol C/m^2^/day), while sulfate-dependent methane oxidation became dominant. Omics data identified the predominant methane-oxidizing bacteria *Methyloprofundus* and QPIN01 (relative abundances of 7%–24%) as the potential mediators of nitrate/nitrite-coupled methane oxidation in the early-stage seep sediments. Consistent with the absence of N_2_O-reduction gene *nosZ* in these lineages, simultaneous nitrate and methane amendments significantly stimulated net N_2_O production by 14%–46% in the early-stage seep sediments. This increase correlated strongly with the potential nitrate/nitrite-coupled methane oxidation rate and was estimated to offset 41% ± 9% of the climate benefit gained through methane oxidation. These findings revealed the previously unrecognized successional dynamics of dominant methane oxidation pathways and necessitate the incorporation of nitrate/nitrite-coupled methane oxidation into assessments of greenhouse-gas release from these globally distributed ecosystems.

**IMPORTANCE:**

Cold seep biota are widely regarded as a critical “biofilter” that restricts seafloor methane emission, mainly through coupled methane and sulfur cycling. This study refines this prevailing view by demonstrating a major shift in the dominant methane oxidation pathway during seep ecosystem development. Contrasting to the dominance of sulfate-coupled methane oxidation in mature seep systems, the early-stage seep is dominated by nitrate/nitrite-coupled methane oxidation, a process primarily mediated by methane-oxidizing bacteria. Crucially, these organisms possess a truncated denitrification pathway that leads to the production of nitrous oxide (N_2_O), a greenhouse gas with nearly 10 times the warming potential of methane. The resulting net N_2_O production thereby substantially offsets the climate benefit achieved by methane consumption. This study highlights the dual role of cold seep microbiota in regulating climate-active gases, underscoring that accurate assessment of their environmental impact requires a holistic understanding of temporal changes in coupled carbon and nitrogen cycles.

## INTRODUCTION

Marine cold seeps are globally distributed ecosystems where methane oxidation, mainly through coupling with sulfate reduction, forms a microbial “filter” that restricts the release of methane from its vast subseafloor reservoir (1–3). Despite occupying less than 0.05% of the continental slope areas, cold seep biota can locally consume up to 100% of the ascending dissolved methane, mediating 10%–20% of the total methane oxidation along global continental slopes (4, 5). By effectively converting methane to CO_2_, cold seep biota function as globally significant “gatekeepers” that mitigate the release of methane, of which the global warming potential (GWP) is 27–30 times that of CO_2_ on a 100-year scale (6).

Besides sulfate, methane oxidation can be coupled to other electron acceptors such as nitrate/nitrite (7–9). The anaerobic *Methanoperedens* archaea (ANME-2d) have been shown to be capable of mediating nitrate-dependent methane oxidation using the methyl-coenzyme M reductase (MCR) and membrane-bound nitrate reductase (NAR) (7). In addition, the anaerobic *Methylomirabilis* bacteria (NC10 phylum bacteria) can couple methane oxidation to nitrite reduction through an intra-aerobic methane oxidation pathway (8). This process harnesses nitric oxide dismutase (NOD) to produce N_2_ and O_2_, with the latter serving as the substrate for methane activation to methanol by methane monooxygenase (MMO) (8). However, these lineages are often scarce or even absent in marine cold seeps (10–12), despite geochemical and isotopic evidence indicating that nitrate/nitrite reduction and its potential coupling to methane oxidation may actively occur in these global hotspots of methane cycling (13, 14). This discrepancy points to the involvement of other, yet-unidentified microbial mediators. Alternative candidates may be found among aerobic methane-oxidizing bacteria (MOB). Although traditionally considered obligate aerobes, certain MOB have demonstrated the ability to oxidize methane under extremely low or even undetectable oxygen conditions (15–17). Under such oxygen-limiting conditions, MOB may utilize nitrate or nitrite as alternative electron acceptors, although the current paradigm is still that oxygen is required for the initial activation of methane into methanol using the O_2_-dependent MMO (16). In cold seep sediments, oxygen is typically depleted within the uppermost millimeters to centimeters, whereas nitrate/nitrite can penetrate much deeper (18). This geochemical niche could, in principle, support MOB-driven, nitrate/nitrite-coupled methane oxidation. Nevertheless, whether and to what extent MOB actually mediates this process *in situ* in cold seep environments remains an open question.

The nitrate/nitrite-coupled methane oxidation has the potential to produce nitrous oxide (N_2_O), which possesses a GWP ~270 times that of CO_2_ on a 100-year scale (19, 20). However, whether elevated methane fluxes amplify N_2_O production in natural environments is still debated. In terrestrial ecosystems, methane has been reported to enhance N_2_O production by inhibiting the nitrous oxide reductase activity (21), while it was also found to reduce N_2_O emissions by promoting complete denitrification through the supply of electrons and carbon substrates (15). In marine cold seeps, where vast amounts of methane migrate from subsurface to surface sediments, it remains poorly constrained whether, how, and to what extent the increased methane availability stimulates N_2_O production. Moreover, cold seeps are highly dynamic ecosystems with biogeochemical conditions, microbial communities, and faunal assemblages shifting across developmental stages (18, 22, 23). While these changes could substantially alter the coupling between nitrate/nitrite reduction and methane oxidation, the extent to which this coupling varies across the developmental stages of cold seep and its consequent impact on N_2_O production remains largely unknown.

**Fig 1:**
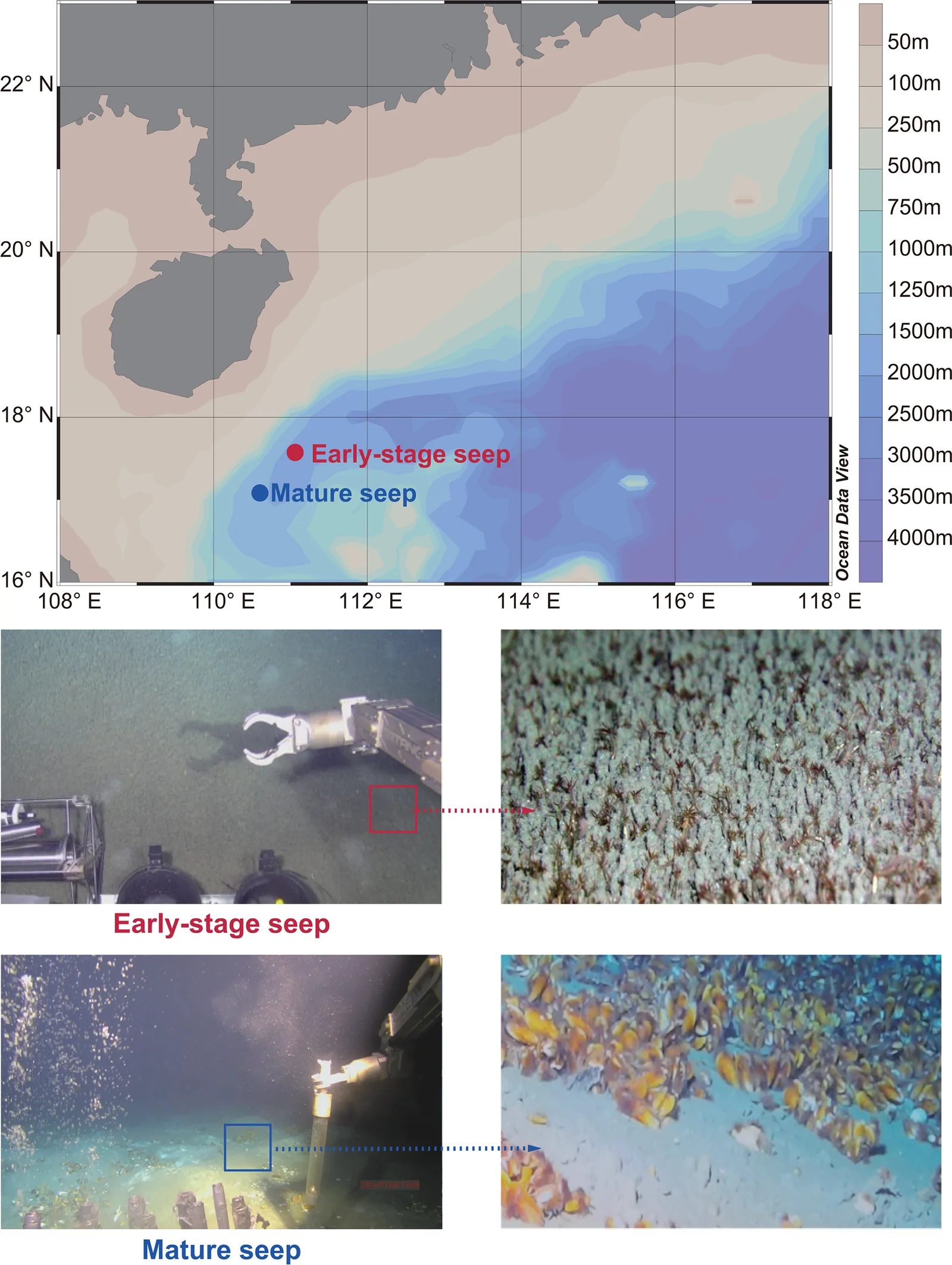
The schematic map of sampling locations and representative seafloor images of the early-stage and mature seep. The early-stage seep ecosystem was dominated by burrowing polychaetes, whereas the mature seep ecosystem was dominated by sessile epifauna (mainly bathymodiolin mussels).

To address these knowledge gaps, we investigated the coupling of nitrate/nitrite reduction and methane oxidation in an early-stage seep ecosystem dominated by the burrowing polychaetes, in comparison to a mature seep dominated by the stationary epifauna (bathymodiolin mussels) in the same region of the South China Sea (23, 24) (Fig. 1). We integrated sediment geochemical profiling, ^14^C-isotope labeling, and omics analyses to quantify the process rates and identify the potential microbial mediators of nitrate/nitrite-coupled methane oxidation and the subsequent N_2_O production. We furthermore conducted manipulative experiments to evaluate the effect of enhanced methane supply on N_2_O production in sediments across the developmental stages of cold seep. Our results reveal the contributions of nitrate/nitrite-coupled methane oxidation across the developmental stages of cold seeps, underscoring the previously underappreciated importance of MOB in regulating the cycling of three greenhouse gases, CH_4_, N_2_O, and CO_2_, in marine cold seeps.

## RESULTS

### Geochemical profiles

**Fig 2:**
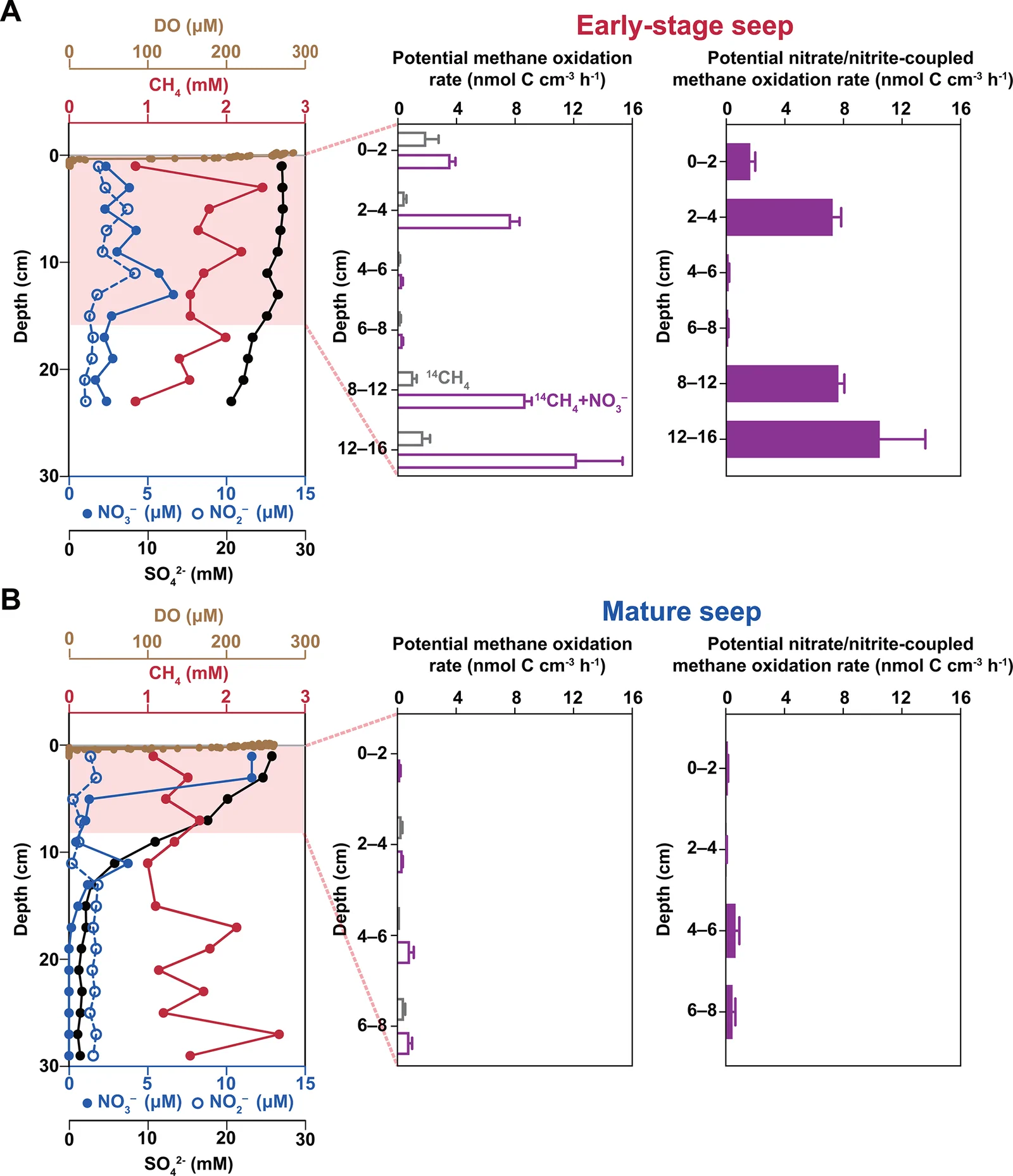
Geochemical profiles and potential rates of nitrate/nitrite-coupled methane oxidation quantified by ^14^C-labeling in (**A**) early-stage and (**B**) mature seep sediments. The potential nitrate/nitrite-coupled methane oxidation rate was calculated as the potential methane oxidation rate of treatment (^14^CH_4_ + NO_3_^−^) minus that of treatment (^14^CH_4_). All of the rate measurements were conducted in triplicates, with filled color bars and error bars showing the mean values and standard deviations, respectively.

Sediment cores were collected from cold seeps at two distinct developmental stages: an early-stage, polychaete-dominated seep and a mussel-dominated, mature seep. Depth-resolved geochemical profiles, including microelectrode measurements of dissolved oxygen and porewater analyses of methane, nitrate/nitrite, and sulfate, revealed the availability of substrates necessary to support nitrate/nitrite-coupled methane oxidation in both habitat types (Fig. 2). At both habitats, dissolved oxygen (DO) was depleted to below the detection limit (0.3 µM) within the upper 0.5 cm of the sediments. Methane concentrations were significantly elevated in seep sediments (early-stage seep: 0.8–2.5 mM; mature seep: 0.4–2.7 mM) compared to the background non-seep sediments (<0.01 mM, Fig. S1). In the early-stage seep, total concentrations of nitrate and nitrite peaked at 10–14 cm (8–10 µM), corresponding to a local minimum of methane concentration. In contrast, the mature seep sediments exhibited their primary nitrate/nitrite peak (~13 µM) in the upper 4 cm, with concentrations decreasing sharply to <2 µM by 8 cm depth, followed by a secondary nitrate/nitrite peak (~4 µM) at 12 cm. In the early-stage seep, sulfate concentration remained relatively constant in the upper 13 cm (26.7 ± 0.7 mM) before decreasing to ~21 mM at 23 cm. In comparison, sulfate concentration showed a sharper downcore decrease in the mature seep sediments, where sulfate decreased from ~28 mM at 1 cm to ~2 mM at 17 cm. Based on these profiles, the depth intervals encompassing the major nitrate/nitrite peaks (early-stage seep: 0–16 cm; mature seep: 0–8 cm) were selected for the subsequent quantification of potential nitrate/nitrite-coupled methane oxidation rates.

### Potential rates of nitrate/nitrite-coupled methane oxidation

Potential rates of methane oxidation coupled to nitrate/nitrite reduction were measured by incubating homogenized sediment sections with ^14^C-labeled methane and nitrate in the absence of oxygen (Fig. 2). In all incubation layers, the simultaneous addition of ^14^CH_4_ and NO_3_⁻ resulted in higher ^14^CO_2_ production compared to amendments with ^14^CH_4_ alone, indicating the occurrence of nitrate/nitrite-coupled methane oxidation. The calculated potential rates differed substantially across the developmental stages of the cold seep. In the early-stage seep, the potential nitrate/nitrite-coupled methane oxidation rate ranged from 0.14 ± 0.03 to 10.48 ± 3.12 nmol C cm^−3^ h^−1^ (Table S1). In the mature seep, the potential rates were substantially lower, ranging from 0.06 ± 0.01 to 0.67 ± 0.26 nmol C cm^−3^ h^−1^.

### Potential mediators of nitrate/nitrite-coupled methane oxidation

**Fig 3:**
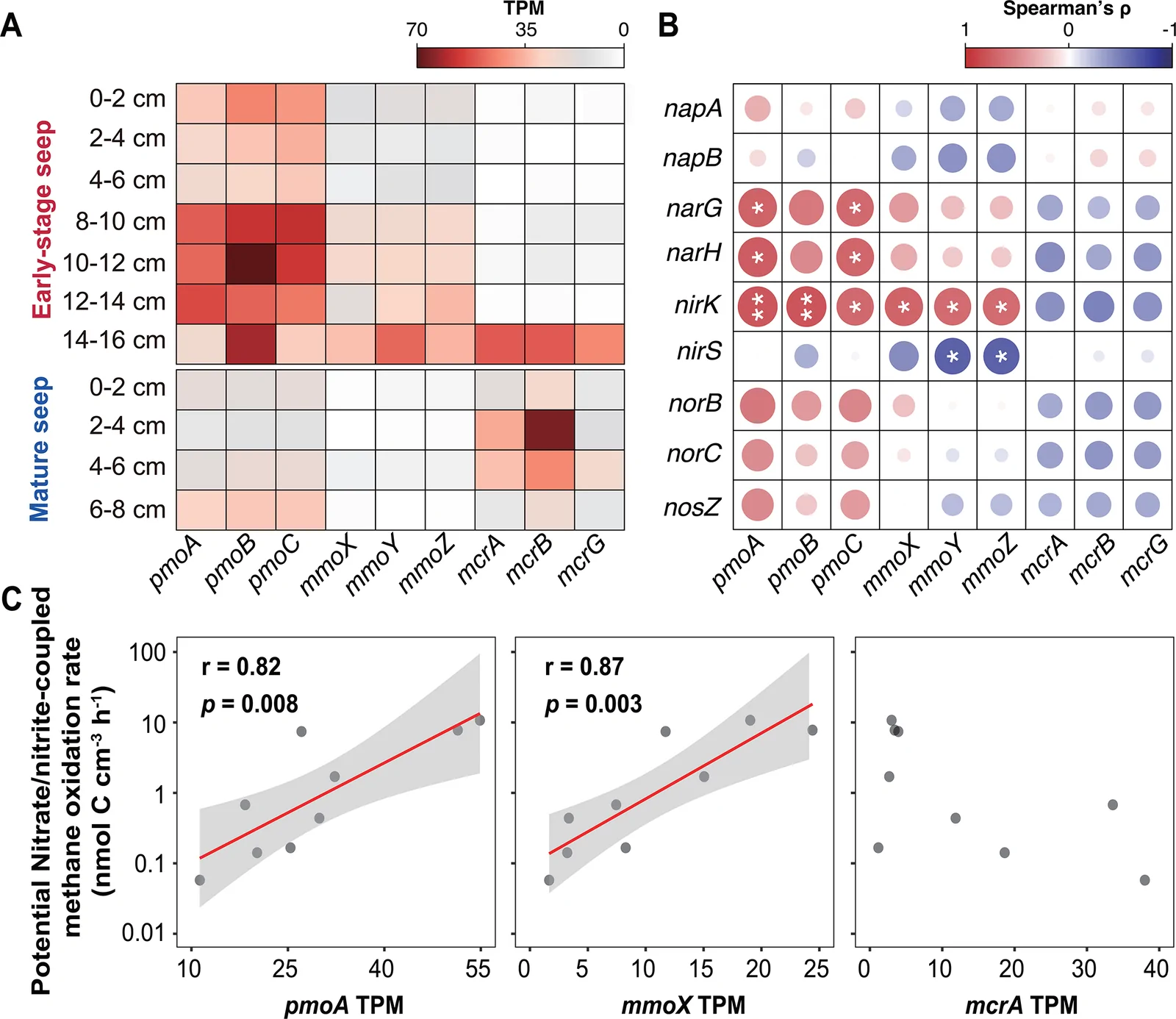
Functional gene evidence for the potential coupling of methane oxidation with nitrate/nitrite reduction. (**A**) Values of transcripts per million (TPM) of methane oxidation genes in surface sediments of the early-stage and mature seeps. (**B**) Spearman correlations between TPM values of methane oxidation genes and nitrate/nitrite reduction genes. The asterisk indicates the statistical significance of Spearman correlation (*: *P* < 0.05; **: *P* < 0.01). (**C**) Pearson correlations of the potential nitrate/nitrite-coupled methane oxidation rates with TPM values of genes associated with different methane oxidation pathways.

Metagenomic analysis revealed a functional linkage between methane oxidation and nitrate/nitrite reduction in the cold seep sediments (Fig. 3). In the early-stage seep sediments, genes encoding methane monooxygenases (*pmoABC* and *mmoXYZ*) were relatively more abundant than those encoding methyl-coenzyme M reductase complex (*mcrABC*), a pattern that reversed in the mature seep sediments (Fig. 3A). Notably, the relative abundances of genes encoding methane monooxygenases (*pmoABC* and *mmoXYZ*) correlated positively with denitrification genes (*narG*, *nirK*, and *norB*) and with the measured potential rates of nitrate/nitrite-coupled methane oxidation (Fig. 3B and C), suggesting the potential key role of MOB in mediating nitrate/nitrite-coupled methane oxidation in the early-stage seep sediments.

**Fig 4:**
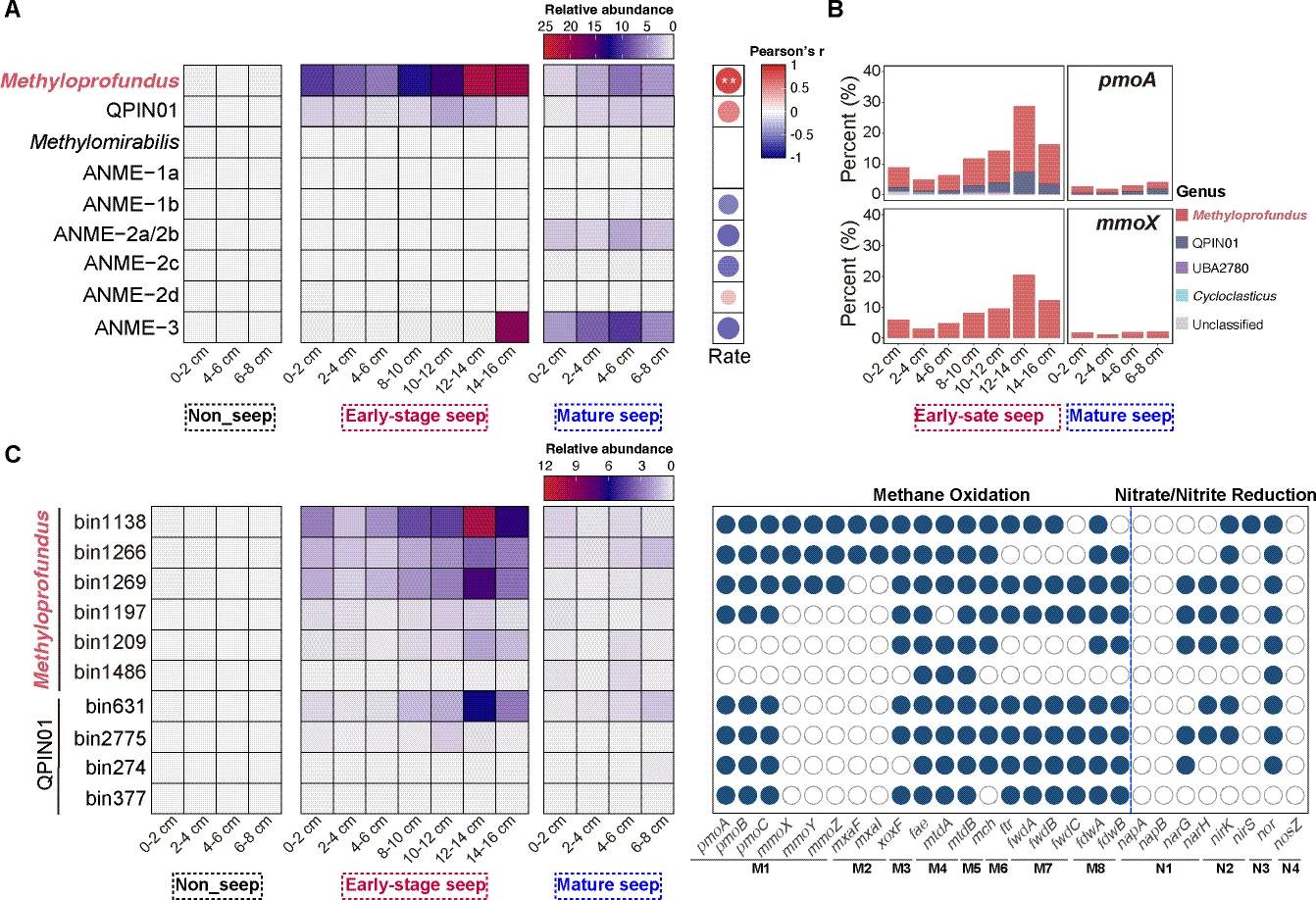
Community and MAG analyses to identify the potential mediators of nitrate/nitrite-coupled methane oxidation. (**A**) Relative abundances of methanotrophs based on the amplicon sequencing of 16S rRNA genes (left panel) and their Pearson correlations with the potential nitrate/nitrite-coupled methane oxidation rate (right panel). Genera *Methylomirabilis* and ANME-1a were not detected, and thus, their Pearson correlations with the potential nitrate/nitrite-coupled methane oxidation rate could not be calculated. (**B**) Taxonomic assignment of MAGs that possessed the methane oxidation genes (top: *pmoA* gene; bottom: *mmoX* gene). (**C**) Relative abundance of dominant MOB MAGs (genera *Methyloprofundus* and QPIN01) based on metagenomic sequencing (left panel) and metabolic reconstruction of the nitrate/nitrite reduction and methane oxidation pathways in these MAGs (right panel). Methane oxidation is conducted sequentially by (M1) *pmoABC* or *mmoXYZ*, (M2) *mxaFI* or *xoxF*, (M3) *fae*, (M4) *mtdAB*, (M5) *mch*, (M6) *ftr,* (M7) *fwdABC*, and (M8) *fdwAB*. Nitrate/Nitrite reduction is conducted sequentially by (N1) *napAB* or *narGH*, (N2) *nirS* or *nirK*, (N3) *nor,* and (N4) *nosZ*.

The 16S rRNA gene amplicon sequencing further identified the genera *Methyloprofundus* and QPIN01 of family *Methylomonadaceae* as the dominant MOB and potential mediators of the enhanced nitrate-/nitrite-coupled methane oxidation in the early-stage seep sediments (Fig. 4). The known nitrate/nitrite-dependent methanotrophs (ANME-2d and *Methylomirabilis*) were nearly absent in all sediment samples (<0.1%, Fig. 4A). By contrast, the MOB genus *Methyloprofundus* predominated the early-stage seep communities (6%–21% relative abundances) but was less abundant in the mature seep communities (2%–6%) and nearly absent in the non-seep communities (<0.1%). Known anaerobic methanotrophic lineages (ANME-1, -2, and -3) were also scarce in early-stage seep sediments, except for ANME-3 at the deepest layer (15 cm). Community analysis based on the *pmoA* and *mmoX* genes confirmed the dominance of *Methyloprofundus*, followed by QPIN01, in the MOB communities of the early-stage seep sediments (Fig. 4B). Notably, only the relative abundances of *Methyloprofundus*, but not other methanotrophs, showed strong positive correlations with the potential rates of nitrate/nitrite-coupled methane oxidation (*r* = 0.84, *P* < 0.01; Pearson correlation; Fig. 4A), implicating its engagement in this process.

To further assess the metabolic potential of these taxa, we analyzed six medium- to high-quality *Methyloprofundus* metagenome-assembled genomes (MAGs; completeness 60%–97% and contamination 1%–7%; Fig. 4C). The three most abundant MAGs (bin1138, bin1266, and bin1269) encoded the majority of the genes involved in methane oxidation and partial denitrification (*narG*, *nirS*, *nirK*, or *nor* gene). Similarly, MAGs of the second most abundant MOB lineage, QPIN01, also encoded most of the genes involved in methane oxidation and partial denitrification. Notably, genes encoding different NOR classes (bNOR, cNOR, eNOR, gNOR, nNOR, qNOR, and sNOR) that catalyze N_2_O production are present in most of the *Methyloprofundus* and QPIN01 MAGs (9/10; Table S2). However, the *nosZ* gene for N_2_O reduction was absent in all *Methyloprofundus* and QPIN01 MAGs, indicating a potential for net N_2_O production. This genomic trait was also confirmed by screening 48 publicly available *Methyloprofundus* genomes and MAGs, none of which contained the *nosZ* gene (Table S3).

### Methane-stimulated N_2_O production

**Fig 5:**
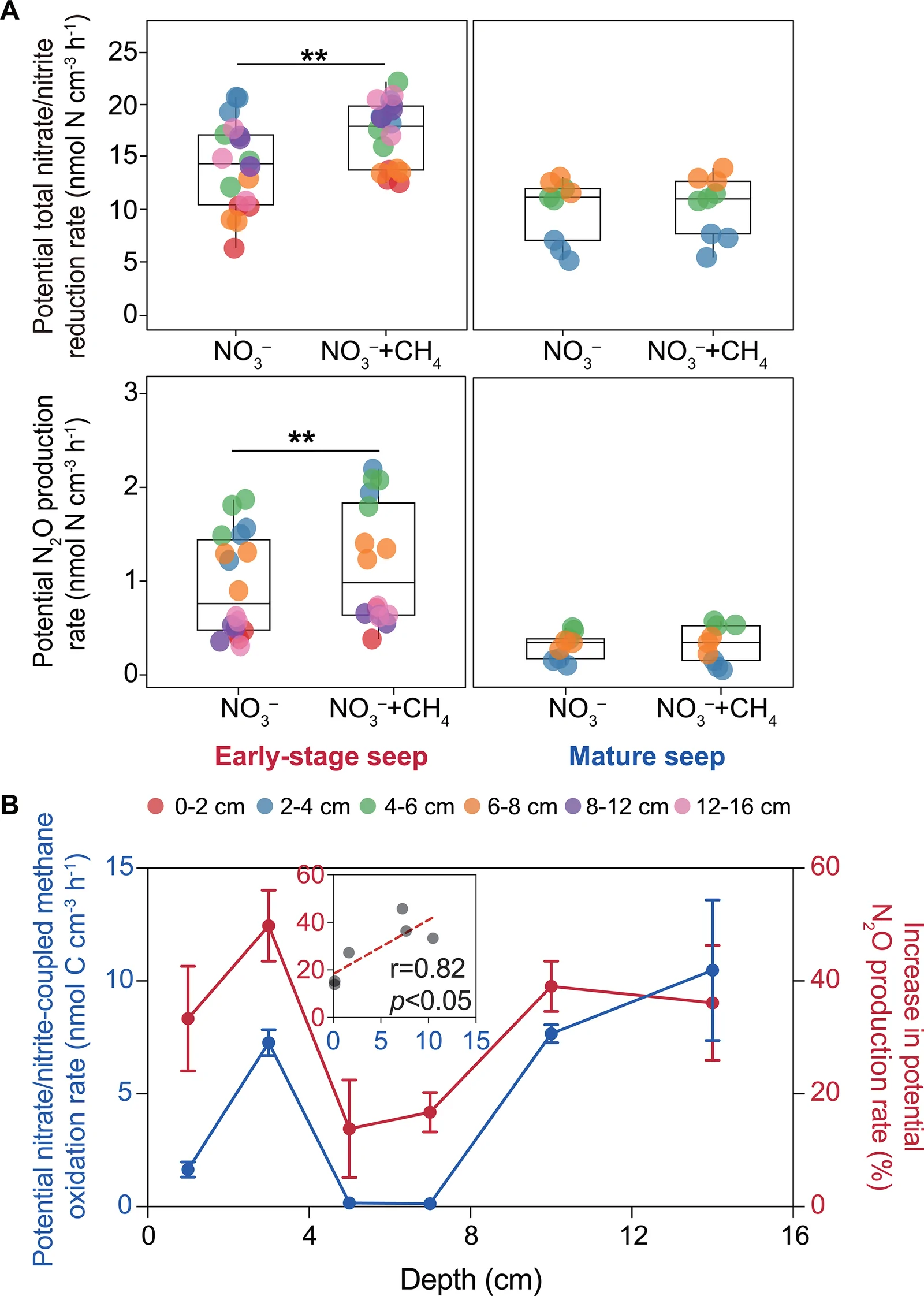
Manipulative experiments to assess the link between nitrate/nitrite-coupled methane oxidation and N_2_O production in early-stage and mature seep sediments. (**A**) The potential total nitrate/nitrite reduction rate (top) and the potential N_2_O production rate (bottom) in treatments with nitrate addition only (NO_3_^–^) and with both nitrate and methane addition (NO_3_^–^ + CH_4_). The asterisk indicates statistical significance (**: *P* < 0.01) based on paired Wilcoxon test. Each data point represents the value derived from an incubation replicate, with triplicate samples for each depth. (**B**) Relationship between the potential nitrate/nitrite-coupled methane oxidation rates and the increases in potential N_2_O production rates (%) in the early-stage seep sediments. The increases in potential N_2_O production rates were calculated as the differences in potential N_2_O production rates between the NO_3_^–^ + CH_4_ treatment and NO_3_^–^ treatment shown in panel **A**. Points represent mean values, and error bars indicate standard deviations (SD); the inset shows the Pearson correlation. Notably, in some cases, the error bars are smaller than the symbols and thus are not visible.

Given the consistent absence of *nosZ* gene in dominant methanotrophs (*Methyloprofundus* and QPIN01) and the high potential rates of nitrate/nitrite-coupled methane oxidation in the early-stage seep sediments, we conducted slurry incubation experiments to test whether the *Methyloprofundus*-dominated, early-stage seep sediments exhibit enhanced N_2_O production (Fig. 5). Compared to controls amended with nitrate only, the simultaneous addition of nitrate and methane significantly stimulated both the nitrate/nitrite reduction and net N_2_O production in the early-stage seep sediments (Fig. 5A; both *P* < 0.01, paired Wilcoxon test). In contrast, methane addition had no significant effect on these processes in mature seep sediments, where the potential nitrate/nitrite-coupled methane oxidation rates were low. Compared to the control group, methane addition increased the net N_2_O production by 14%–46% across different depths of the early-stage seep sediments, and such an increase correlated significantly with the potential nitrate/nitrite-coupled methane oxidation rates (r = 0.82, *P* < 0.05, Pearson correlation), directly linking methanotrophic activity to the potential of enhanced N_2_O yield (Fig. 5B).

## DISCUSSION

### Stage-dependent shift in methane oxidation pathways

Our study reveals a previously unrecognized shift in the dominant methane oxidation pathway across the developmental stages of cold seeps. In the early-stage seep, the depth-integrated (0–16 cm) potential rate of nitrate/nitrite-coupled methane oxidation reached 21.9 ± 3.0 mmol C m^−2^ day^−1^ (Table S1), which is on the same order of magnitude as the depth-integrated total methane oxidation rate (30–60 mmol C m^−2^ day^−1^) previously modeled for the upper 100 cm sediment of the same site (18). Assuming that nitrate/nitrite-coupled methane oxidation was negligible in deeper sediment layers, we conservatively estimated that this process accounts for 36%–73% of the total methane oxidation in the 0–100 cm early-stage seep sediments. This suggested that methane oxidation coupled to nitrate/nitrite reduction constituted a critical sink for methane in the polychaete-dominated, early-stage cold seep. In stark contrast, its contribution diminished to only ~1% (0.62 ± 0.16 mmol C m^−2^ day^−1^) in the mature seep sediments dominated by stationary, epifauna (bathymodiolin mussels), where sulfate-dependent methane oxidation is presumed to dominate, given the rapid depletion of sulfate within the upper 15 cm sediments, consistent with the prevalence of sulfate-driven methane oxidation in typical mature seep systems (25–27). This process transition was further supported by a pronounced increase in the relative abundance of anaerobic methanotrophic archaea (ANME), which typically form syntrophic consortia with sulfate-reducing bacteria (28), from negligible levels in early-stage seep sediments to 7%–14% in mature seep sediments (Fig. 4A).

We attribute the prominence of nitrate/nitrite-coupled methane oxidation in the early-stage seep sediments primarily to intense bioturbation induced by the burrowing Spionidae polychaetes (Fig. 1 and Fig. S2), which facilitated the transport of dissolved oxygen and nitrate from overlying seawater into the bioturbated sediments (18, 29, 30). The downward-transported dissolved oxygen was rapidly depleted by various oxygen-consuming processes, likely including the initial activation of methane into methanol via methane monooxygenase, and thus fell below the detection limit of conventional oxygen microsensor across most sediment depths (>0.5 cm, Fig. 2). Under such oxygen-limited conditions, subsequent methane oxidation process may preferentially be coupled with nitrate/nitrite reduction, given the higher energy yield of nitrate/nitrite over sulfate as electron acceptors (31). To cross-check, we further developed a simplified reaction-transport model to estimate the transport and reaction fluxes of nitrate/nitrite (see Materials and Methods for details). Considering the ventilation rate and density of the dominant faunal group in the early-stage seep sediments, the Spionidae polychaetes, the transport flux of nitrate/nitrite from overlying water (~51.3 µmol N L^−1^, mainly nitrate) into sediments reached 55.6 ± 17.7 mmol N m^−2^ day^−1^, suggesting a sufficient amount of nitrate/nitrite to sustain the high potential rates of nitrate/nitrite-coupled methane oxidation we measured. Despite this high influx, the nitrate/nitrite concentrations in sediments were low (~5 µmol N L^−1^), suggesting a fast nitrate/nitrite consumption with modeled rates (50.2 ± 16.0 mmol N m^−2^ day^−1^) matching the potential total nitrate/nitrite reduction rate measured in our incubation systems (55–68 mmol N m^−2^ day^−1^). Applying the fraction of nitrate/nitrite allocated to methane oxidation (52%–100%) based on our incubation experiments, the modeled nitrate/nitrite-coupled methane oxidation ranged from 6.5–16.3 to 12.5–31.3 mmol N m^−2^ day^−1^, generally aligning with our tracer-based potential rates (21.9 ± 3.0 mmol C m^−2^ day^−1^). Using the lower end of this estimate, we estimated that nitrate/nitrite-coupled methane oxidation accounted for a notable fraction (11%–54%) of the total methane oxidation in the early-stage seep sediments, generally matching the fraction of 36%–73% estimated by tracer-based potential rates. These results reveal the overlooked, important contribution of nitrate/nitrite-coupled methane oxidation in the early-stage seep system, consistent with the high abundance of MOB with metabolic potential of nitrate/nitrite-coupled methane oxidation (discussed in the next paragraph), providing new insights into the dynamics of carbon and nitrogen coupling across the developmental stages of cold seep (18).

### MOB as prime candidates mediating nitrate/nitrite-coupled methane oxidation

The high potential rates of nitrate/nitrite-coupled methane oxidation measured in the early-stage seep were most likely mediated by the aerobic MOB (Fig. 3 and Fig. 4). The MOB genera *Methyloprofundus* and QPIN01 predominated in surface sediments of the early-stage seep (7%–24% of the total 16S rRNA gene sequences), where the known nitrate/nitrite-dependent methanotrophs (ANME-2d and *Methylomirabilis*), as well as other known methanotrophs, were all scarce or absent. The abundances of *Methyloprofundus* and QPIN01 generally increased with depth and maximized in the 12–16 cm layers, precisely where the highest potential rates of nitrate/nitrite-coupled methane oxidation were measured (Fig. 2A). The positive correlations of the potential nitrate/nitrite-coupled methane oxidation rates with *pmoA* and *mmoX* gene abundances and with the relative abundances of *Methyloprofundus* further support the involvement of MOB *Methyloprofundus* in nitrate/nitrite-coupled methane oxidation (Fig. 2C and Fig. 3A). These ecological correlations are moreover supported by genomic evidence. Dominant *Methyloprofundus* MAGs possess the genetic machinery for both methane oxidation and partial denitrification pathway (Fig. 3C). A broader genomic survey of 54 *Methyloprofundus* genomes (6 from this study and 48 public) revealed a high prevalence of *narG* (47/54) and *nirK* (52/54), a low incidence of *nirS* (5/54) and *norB* (22/54), and the absence of *napA* (0/54) and *nosZ* (0/54) (Table S3). This genetic architecture indicates a consistent capacity for truncated denitrification potential of *Methyloprofundus*, aligning with the demonstrated denitrifying activity among diverse isolates and enrichment cultures of other MOB (32–34). The specific suite of denitrification genes also informs the ecological adaptation of *Methyloprofundus*. The dominance of *narG* and *nirK*—genes associated with denitrifiers that are generally adapted to hypoxic/anoxic conditions—over the oxygen-tolerant *napA* and *nirS* genes (35–38) indicates the specialization of *Methyloprofundus* for low-oxygen conditions. Furthermore, the membrane-bound *narG* has been shown with higher *in vitro* enzymatic activity and greater ATP yield compared to the periplasmic *napA* (39, 40), likely maximizing energy conservation and facilitating the ecological success of *Methyloprofundus* in the nitrate-replete, low-oxygen sediments of the early-stage seep.

### Role of nitrate/nitrite-coupled methane oxidation in N_2_O production

Our integrated results show that nitrate/nitrite-coupled methane oxidation, likely mediated by MOB, represents a previously underappreciated source of N_2_O in marine cold seeps. The consistent absence of the *nosZ* gene in dominant MOB *Methyloprofundus* and QPIN01 suggests a genomic potential for net N_2_O production (Fig. 4C and Table S3). This genetic predisposition was functionally verified by our manipulative experiments. Compared with controls amended with nitrate only, simultaneous methane and nitrate addition significantly stimulated net N_2_O production by 14%–46% in the *Methyloprofundus*-dominated, early-stage seep sediments (Fig. 5 and Table S4). This increase in N_2_O production correlated strongly with the measured potential nitrate/nitrite-coupled methane oxidation rates (Fig. 5B), directly linking the nitrate/nitrite-coupled methane oxidation activity to enhanced N_2_O yield. Given that N_2_O and CH_4_ have a warming potential ~270 and ~27 times greater than CO_2_, we furthermore estimated that the CO_2_ equivalent of enhanced N_2_O production (234.9 ± 40.9 mmol C m^−2^ day^−1^) accounts for 41% ± 9% of that derived from methane oxidation to CO_2_ via nitrate/nitrite-coupled methane oxidation (568.1 ± 78.9 mmol C m^−2^ day^−1^). It suggests that a considerable fraction of the climate benefit gained from the nitrate/nitrite-coupled methane oxidation could be offset by the concurrent N_2_O production. In contrast, the lack of a significant increase in N_2_O production in the mature seep sediments aligns with the minimal activity of nitrate/nitrite-coupled methane oxidation and the ecological shift toward sulfate-dependent methane oxidation. Given the prevalence of MOB in cold seep sediments (3) and the widespread absence of *nosZ* in their genomes (361 out of 371 genomes screened; Fig. S3 and S4), a truncated denitrification pathway that culminates in net N_2_O production may be a common trait of MOB in cold seep environments. Collectively, these findings highlight the stage-specific dynamics of MOB and their dual climate effect, which should be incorporated into biogeochemical models of cold seep ecosystems (6).

## MATERIALS AND METHODS

### Study sites

The study area is located in the Qiongdongnan Basin, northern South China Sea. We focused on two distinct types of cold seeps: an early-stage seep and a mature seep ecosystem. The early-stage seep (111.06°E, 17.63°N; water depth: 1,758 m) is subject to a recently activated methane seepage (18, 41). Within the seepage area, dense faunal populations dominated by Spionidae polychaetes colonized the soft sediments (18, 24), while typical seep epifauna and carbonate crusts were absent (Fig. 1), confirming its early-stage status. At this site, three sediment push cores were collected using the remotely operated vessel (ROV) *“Haima”* in 2024, from a site of active methane seepage (sediment methane concentration: 0.8–2.5 mM, Fig. 2A) and from an adjacent non-seep background site (methane concentration: <0.01 mM, Fig. S1). For comparison, a mature seep site was also sampled within the same basin (110.6°E, 17.14°N; water depth: 1,521 m) in 2024. This site is characterized by extensive mussel beds (*Bathymodiolus* sp.) and carbonate crusts (Fig. 1), matching the features of a mature seep ecosystem under long-term seepage activity (2, 23, 42). Three sediment push cores were collected from an active seep area at this site, where porewater methane concentrations ranged from 0.4 to 2.7 mM (Fig. 2B).

### Sample collection and geochemical analysis

Upon retrieval, sediment cores were rapidly transferred to a 4°C cool room. For each sampling site, one sediment core was immediately used for Unisense micro-profiling measurements of dissolved oxygen (O_2_, Clark-type sensor with guard cathode; detection limit: 0.3 µM) at 4°C. Afterward, sediment samples were taken by extruding the sediments out of the core liner. Another sediment core was applied for porewater extraction at 2 cm depth intervals, using the Rhizon samplers (0.2 μm pore size, Rhizosphere, the Netherlands). Samples for different analytical purposes were taken and stored under suitable conditions (Text S1). As previously described, nitrate and nitrite concentrations were quantified using the continuous-flow AutoAnalyzer (AA3, SEAL); sulfate concentration was measured by the ion chromatography (ICS-5000+/900, Agilent), while dissolved CH_4_ concentration was measured by the gas chromatography (GC-2030, Shimadzu) equipped with barrier ionization (BID) and flame thermionic (FTD) detectors (18). The detection limits for nitrate and nitrite were 0.1 and 0.04 μM, respectively, with a precision better than 0.4% and 1.2%.

### Quantification of nitrate/nitrite-coupled methane oxidation

Radiotracer assays were conducted to determine the potential nitrate/nitrite-coupled methane oxidation rate (43–45). Briefly, homogenized sediment slurries were amended with (i) ^14^CH_4_ and (ii) ^14^CH_4_ + NO_3_^−^. Triplicate sediment samples and a killed control were taken at the same depth. Potential methane oxidation rates of different treatments were quantified according to the production of ^14^CO_2_ during incubation. Subsequently, the potential nitrate/nitrite-coupled methane oxidation rate was calculated by subtracting the potential methane oxidation rate of treatment ii from the potential rate of treatment i (46). Additional methodological details are provided in Supplemental Methods in Text S2.

### Manipulative experiments

A sediment slurry assay for quantifying the potential N_2_O production rates was used as described previously (47). To evaluate the effect of methane on N_2_O production, two treatments were implemented: (i) nitrate addition only (NO_3_^–^) and (ii) both nitrate and methane addition (NO_3_^–^ + CH_4_). The final concentration of NO_3_^–^ in each vial was ~100 µM. For incubations involving CH_4_, artificial seawater adjusted to *in situ* salinity was prefiltered through a 0.2 μm membrane filter (Millipore, Merck) and then equilibrated to saturation with CH_4_. At the beginning of incubation, 3 mL of the culture supernatant was aseptically withdrawn and replaced with an equal volume of CH_4_-saturated artificial seawater, reaching a final dissolved methane concentration of ~0.5 mM, comparable to the lower end of *in situ* methane concentrations. Dissolved N_2_O was quantified by a headspace-equilibration method (48). Briefly, ultrahigh-purity helium was introduced into the vial to create a headspace after incubation. Headspace N_2_O was then analyzed by gas chromatography (Shimadzu GC-2014; detection limit of 0.1 ppb). Dissolved concentrations were calculated using Bunsen and solubility coefficients (49). The production rates of N_2_O during incubations were calculated using the equation reported by Hou et al. (47). The changes in nitrate and nitrite concentrations were measured to determine the potential total nitrate/nitrite reduction rate. Additional methodological details are provided in Supplemental Methods in Text S3.

### Reaction-transport modeling

A simplified reaction-transport box model was applied to estimate the nitrate/nitrite-coupled methane oxidation fluxes in early-stage seep sediments. Given the strong bioturbation activity observed, we assumed that nitrate/nitrite was mainly supplied to sediment via the strong bioturbation by fauna (Fig. S2) rather than the slow diffusion. The fauna-mediated ventilation flux was calculated as follows:


$$J_{q}=q\times N$$

where *J*_*q*_ (mmol N m^−2^ day^−1^) is the ventilation flux and *q* is the ventilation rate of the dominant fauna (Spionidae polychaete) in our system, with previously reported mean values of 3.6 mL h^−1^ individual^−1^ (*n* = 41) (50). *N* is the density of Spionidae polychaetes in the early-stage seep sediments (12,557 ± 3,997 individuals m^−2^, *n* = 5) (18).

The consumption rate of nitrate and nitrite was calculated as follows:


$${NO}_{x\_c}=J_{q}\times \left({NO}_{x\_bw}-{NO}_{x\_pw}\right)$$

where NO_*x*_bw_ is the nitrate/nitrite concentration in the bottom water (~51.3 µmol N L^−1^), NO_*x*_pw_ is the average nitrate/nitrite concentration in the porewater (~5 µmol N L^−1^), and NO_*x_c*_ is the consumption rate of nitrate and nitrite.

The nitrate/nitrite-coupled methane oxidation flux was further estimated by assuming that 52%–100% of the nitrate/nitrite was used for methane oxidation based on our incubation experiments and considering the possible stoichiometries for methane oxidation coupled to nitrate/nitrite reduction: (i) 5CH_4_ + 8NO_3_^–^ + 8H^+^ → 5CO_2_ + 4N_2_ + 14H_2_O; (ii) 3CH_4_ + 8NO_2_^–^+ 8H^+^ → 3CO_2_ + 4N_2_ + 10H_2_O; (iii) CH_4_ + 2NO_3_^–^+ 2H^+^ → CO_2_ + N_2_O + 3H_2_O; and (iv) CH_4_ + 4NO_2_^–^+ 4H^+^ → CO_2_ + 2N_2_O + 4H_2_O.

### DNA extraction and sequencing

DNA was extracted from about 0.5 to 2.0 g of sediment samples as described previously (51). For 16S rRNA gene high-throughput sequencing, the V4 region of 16S rRNA genes was amplified using the primer pair 515F/806R (52), and sequencing was performed at Majorbio (Shanghai, China) using the Illumina MiSeq platform (Illumina, USA). For metagenomic sequencing, the library was constructed using the TruePrep DNA Library Prep Kit (Vazyme, Nanjing, China), and paired-end sequencing with 150-bp length was performed at BGI Genomics (Shenzhen, China) using the Illumina NovaSeq platform (Illumina, USA).

### 16S rRNA gene high-throughput sequencing analysis

The raw reads of the V4 region of the 16S rRNA gene were processed and analyzed using the QIIME 2 platform v2020.11 (53). The primers and adapters were first trimmed out using Cutadapt v3.1 (54). Raw sequences were then processed using DADA2 (55), including quality filtering, denoising, paired-end sequence merging, chimera filtering, and producing amplicon sequence variants (ASVs) and an ASV Table. Taxonomy was assigned using q2-feature-classifier (a scikit-learn naive Bayes machine-learning classifier) (56), with Silva database release 138 (57). Multiple sequence alignment and phylogenetic tree construction were performed using the QIIME 2 plugin q2phylogeny (align-to-tree-mafft-iqtree). Unassigned sequences, singletons, and sequences affiliated with eukaryotes were discarded.

### Metagenomic analysis

Quality filtering and adapter removal were performed with Trimmomatic (58), and high-quality reads were assembled *de novo* using MEGAHIT (59), retaining contigs ≥ 2 kb. Gene prediction and functional annotation were conducted with Prodigal (60) and Kofamscan (61) against the KEGG database (62), with gene abundances normalized as TPM (63). Metagenome-assembled genomes were reconstructed by integrating the binning results from MetaBAT2 (64), MaxBin (65), and CONCOCT (66). MAGs were dereplicated with DAS Tool (67) and refined using RefineM (68). All refined MAGs with completeness >50% and contamination <10% were dereplicated using dRep (69) to obtain species-level representative genomes. Taxonomic classification of MAG was performed using the GTDB-Tk (70), specifically the classify_wf workflow for annotation based on the Genome Taxonomy Database (71). The quality of MAG was evaluated with CheckM1 (72). Prodigal was used to predict protein-coding genes for each MAG, and the proteome of each MAG was annotated using Kofamscan (61) against the KEGG database (62). Nitric oxide reductase (Nor) was also classified according to protein class (i.e., bNOR, cNOR, eNOR, gNOR, nNOR, qNOR, and sNOR) (73). The relative abundances of MAGs were estimated by mapping reads to dereplicated genomes using CoverM (74). Additional methodological details are provided in Supplemental Methods in Text S4.

### Statistics

Statistical analyses were performed in R (V4.5.3; www.r-project.org), and all figures were generated by the “ggplot2” R package or GraphPad Prism 10 (GraphPad Software Inc., San Diego, CA, USA). Differences between the two groups were evaluated using the Wilcoxon test. Pearson correlation coefficients were used to evaluate the relationships between potential nitrate/nitrite-coupled methane oxidation rates and other factors. Spearman correlation coefficients were used to evaluate relationships among TPM values of different genes.

## Data Availability

All sequencing data are available online through the NCBI database under the project number PRJNA1313825.

## SUPPLEMENTAL MATERIAL

Supplemental Material mbio.01446-26-s0001.pdf Supplemental methods, figures, and tables.

ASM does not own the copyrights to Supplemental Material that may be linked to, or accessed through, an article. The authors have granted ASM a non-exclusive, world-wide license to publish the Supplemental Material files. Please contact the corresponding author directly for reuse.
