# Supplementary material for: Nitrate/nitrite-driven methane oxidation enhanced N2O production in marine cold seeps
Source: mBio. 2026 Jul 31;17(9):e01446-26. doi: 10.1128/mbio.01446-26 (PMC13556223; doi:10.1128/mbio.01446-26)
Supplement: Supplementary file 1 [file mbio.01446-26-s0001.pdf]

Supplementary Information for

Nitrate/nitrite-driven Methane Oxidation Enhanced N_2O Production in Marine Cold Seeps

Weikang Sui[1, #], Longhui Deng[1, #], Bing-Zheng Wu[2], Guang-Chao Zhuang[2], Zian Tong[1], Ningyuan Lu[1], Danyue Huang[1], Zheng Xiong[1], Peng Guo[2], Xianbiao Lin[2], Yanwei Wang[1], Liang Dong[1], Fengping Wang[1, *]

[1]*State Key Laboratory of Submarine Geoscience; Key Laboratory of Polar Ecosystem and Climate Change, Ministry of Education; Shanghai Key Laboratory of Polar Life and Environment Sciences; and School of Oceanography, Shanghai Jiao Tong University, Shanghai 200030, China.*

[2]*Frontiers Science Center for Deep Ocean Multispheres and Earth System, and Key Laboratory of Marine Chemistry Theory and Technology, Ministry of Education, Ocean University of China, Qingdao 266100, China.*

[#]These authors contributed equally to this work.

*Corresponding author: Fengping Wang

Email: fengpingw@sjtu.edu.cn

This includes:

Supplemental Text S1 to S4

Supplemental Figure S1 to S3

Supplemental Table S1 to S3

Text S1. Sampling protocol

Upon retrieval, sediment cores were immediately transferred to 4 °C cool room. One sediment core was used for micro-profiling analyses of dissolved oxygen (DO) and porewater extraction. Porewater was extracted from the same core at 2 cm depth intervals using the Rhizon samplers (0.2 μm pore size, Rhizosphere, Netherland) and preserved at −20 °C for subsequent geochemical analyses. Sediment subsamples were collected from another parallel core for methane quantification and microbial analysis. Sediment used for methane measurement was promptly taken using autoclaved, tip-cut syringes and injected into 20 mL headspace vials pre-filled with 2 M NaOH solution. The sediment samples for microbial analyses were taken using autoclaved tip cut-off syringes and preserved at −80°C. Sediment samples used for measuring potential nitrate/nitrite-coupled methane oxidation rate and potential N_2O production rate were collected from another parallel cores. Sediment was sectioned at 2–4 cm intervals and processed immediately for on-deck incubation experiments to measure potential N_2O production rate. Sediment samples used to measure potential nitrate/nitrite-coupled methane oxidation rate were preserved at 4 °C under anoxic conditions and subsequently subjected to laboratory incubation experiments using ^{14}C isotope-pairing techniques.

Text S2. Sediment incubation for determining potential rates of nitrate/nitrite-coupled methane oxidation

Slurries were made with sediment and artificial seawater with *in situ* salinity with a sediment/seawater volume ratio of 1:7, and the mixture was purged with helium for 30 min and stirred vigorously to make homogenized slurries. Subsequently, the slurries were preincubated at near *in situ* temperature for 2–3 days to remove the residual NO_2^-/NO_3^- and dissolved oxygen (1-3). After pre-incubation, 3 mL sediment slurry was introduced into a modified cut-end Hungate tube. Subsequently, 0.75 mL of culture supernatant was aseptically withdrawn and replaced with an equal volume of $^{12}CH_4$-saturated artificial seawater to maintain methane enrichment. For $^{12}CH_4$-saturated

artificial seawater, artificial seawater adjusted to *in situ* salinity was prefiltered through 0.2 μM membrane filter (Millipore, Merck) and then equilibrated to saturation with $^{12}CH_4$. The Hungate tube was then sealed with a gray chlorobutyl stopper and a screw cap. The slurries were amended with the following treatments: (a) $^{14}CH_4$, (b) $^{14}CH_4$ + NO_3^-. The final concentration of NO_3^- in each vial was approximately 200 μM. This concentration is higher than the concentrations measured in porewater (<10 μM), considering that bioturbation strongly promoted the transport of nitrate into sediments under *in situ* condition. A 100 μL aliquot of ^{14}C-CH_4 tracer (American Radiolabeled Chemicals, Inc.; 15–22 kBq in sterile Milli-Q water) was injected into each of the four replicate sediment samples (three live samples and one killed control). The final concentration of CH_4 ($^{12}CH_4$ + $^{14}CH_4$) in each vial was ~0.5 mM, which is comparable to the lower-end methane concentrations at cold seep environments (0.4–2.7 mM). It should also be noted that the added methane (~0.5 mM) was more limiting than the added nitrate (~0.2 mM) based on the reported stoichiometric relationship (4-6). Killed controls were injected with 3 mL of 2 mol L^{-1} NaOH prior to $^{14}CH_4$ addition. The slurries were then incubated at *in situ* temperature for 2 days. After incubation, microbial activity was stopped by injecting 3 mL of 2 mol L^{-1} NaOH, with the same manner as killed control (7, 8). The quantification for production of ^{14}C-CO_2 from ^{14}C-CH_4 was performed with acid digestion method (9). Briefly, well-mixed sediment slurries were transferred to a 250 mL glass bottle and purged with air to remove unreacted ^{14}C-CH_4. Then 5 mL of 30% (v/v) sulfuric acid was added to the flask, and liberated $^{14}CO_2$ was trapped into 7 ml glass scintillation vials filled with 3-methoxypropylamin. Potential methane oxidation rates were quantified using Eq. (1):

$$MeO= (^{14}C\text{-}CO_2)/(^{14}C\text{-}CH_4) \times [CH_4] \times \alpha / t \times 8 \quad (1)$$

where MeO is the potential rate of methane oxidation in units of nmol cm^{-3} h^{-1}; (^{14}C-CO_2) is the $^{14}CO_2$ counts (decay per minute, dpm); (^{14}C-CH_4) is the total injected $^{14}CH_4$ (dpm); [CH_4] is the methane concentration in sediment porewater (μM); α is the

carbon isotopic fractionation correction factor (1.01); t is incubation duration in hours; "8" represents slurry dilution factor.

The potential nitrate/nitrite-coupled methane oxidation rate = potential methane oxidation rate of group (b) − potential methane oxidation rate of group (a).

Text S3. Sediment incubation for determining potential N_2O production rates

Slurries were made with sediment and artificial seawater with *in situ* salinity at a sediment/seawater volume ratio of 1:7, and the mixture was purged with helium for 30 min and stirred vigorously to make homogenized slurries. Subsequently, the slurries were preincubated at near in situ temperature for 2–3 days to remove the residual NO_2^-/NO_3^- and dissolved oxygen (1-3). The slurries were amended with the following treatments: (1) NO_3^- and (2) NO_3^- + CH_4. The final concentration of NO_3^- in each vial was ~100 μM. For incubations involving CH_4, artificial seawater adjusted to *in situ* salinity was prefiltered through 0.2 μM membrane filter (Millipore, Merck) and then equilibrated to saturation with CH_4. At the beginning of incubation, 3 mL of culture supernatant was aseptically withdrawn and replaced with an equal volume of CH_4-saturated artificial seawater to maintain methane enrichment. Random measurements of group (2) (n=10) indicated mean CH_4 concentration of ~0.5 mM, which is comparable to the lower-end methane concentrations at cold seep environments (Fig. 2). The slurries were then incubated at *in situ* temperature. At 0 and 24 h of incubation, triplicate vials were sacrificed by the injection of 200 μL of 50% $ZnCl_2$ solution to terminate activity, representing the initial and final samples, respectively.

After incubation, headspace equilibrium technique was used for the dissolved N_2O measurement (11). Briefly, 2 mL of ultrahigh-purity helium gas was injected into each 12 mL vial to replace the water phase and create a headspace. The vials were shaken vigorously for 1 h to equilibrate the gas and liquid phases. Subsequently, the headspace gas was extracted using a syringe and quantified using gas chromatography (Shimadzu GC-14B) with a detection limit of 0.1 ppb for N_2O. The Bunsen solubility coefficient for the determined salinity and temperature after phase equilibration was

used with the determined N_2O in the headspace to estimate the total N_2O concentration in the sediment slurries (12). The potential production rates of N_2O over the incubations were calculated using the following Eq. (2):

$$R = \frac{(N_f - N_i) \times V}{t} \quad (2)$$

where R (nmol cm^{-3} h^{-1}) denotes the potential N_2O production rate, N_i and N_f (nmol mL^{-1}) indicate the total contents of N_2O dissolved in the initial and final slurry samples, respectively, V (mL) is the volume of the incubation vials, and t (h) represents the incubation time.

Also, the changes of nitrate and nitrite concentrations were measured, to determine the potential total nitrate/nitrite reduction rate.

Text S4. Metagenomic analysis

Adapter trimming and low-quality reads removal were performed by Trimmomatic (v0.38) (13). Clean reads from each sample were then assembled *de novo* with MEGAHIT (v1.2.9) (14) using a k-mer size of 4. Contigs shorter than 2,000 bp were excluded. Genes of each contig were predicted by Prodigal (v2.6.3) (15), then annotated by the KEGG database (16) using Kofamscan (v 1.3.0) (17). For each sample, metabolic potential was estimated by TPM (Transcripts Per Million) of genes (18). Binning was performed using MetaBAT2 (v2.12.1) (19), MaxBin (v2.2.7) (20), and CONCOCT (v1.1.0) (21) under default parameters. Metagenome-assembled genomes (MAGs) from each assembly were dereplicated with DAS Tool (v1.1.6) (22) and refined using RefineM (v0.1.2) (23). All refined MAGs with completeness > 50% and contamination < 10% were dereplicated using dRep (v3.5.0) (24) with the parameter --S_ani 0.95 to obtain the species-level representative genomes. Taxonomic classification of the MAGs was performed using the GTDB-Tk classify_wf workflow (v1.6.0) (25) against the GTDB genome database (Release 220) (26). And quality of MAG was evaluated with CheckM1 (v1.2.3) (27). Prodigal was used to predict protein-coding genes for each MAG and the proteome of each MAG was annotated using the same KEGG annotation protocol. Nitric oxide reductase (NOR) was also classified according to protein class

(i.e., bNOR, cNOR, eNOR, gNOR, nNOR, qNOR and sNOR) (28). The relative abundance of the dereplicated MAGs was calculated using CoverM (v0.7.0) (29), which mapped metagenomic sequences to the MAGs with default parameters.

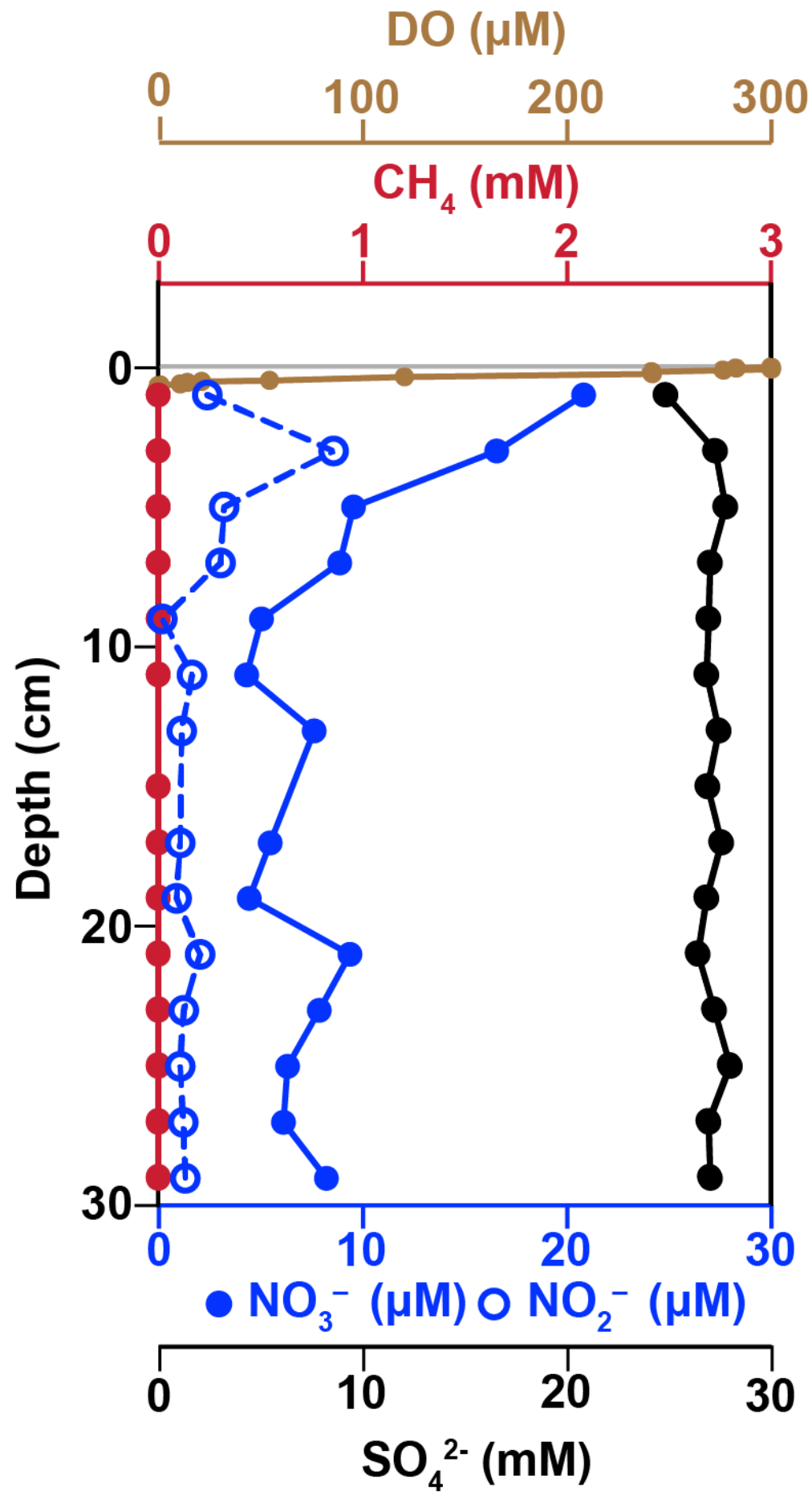

Figure S1. Geochemical profiles of non-seep station.

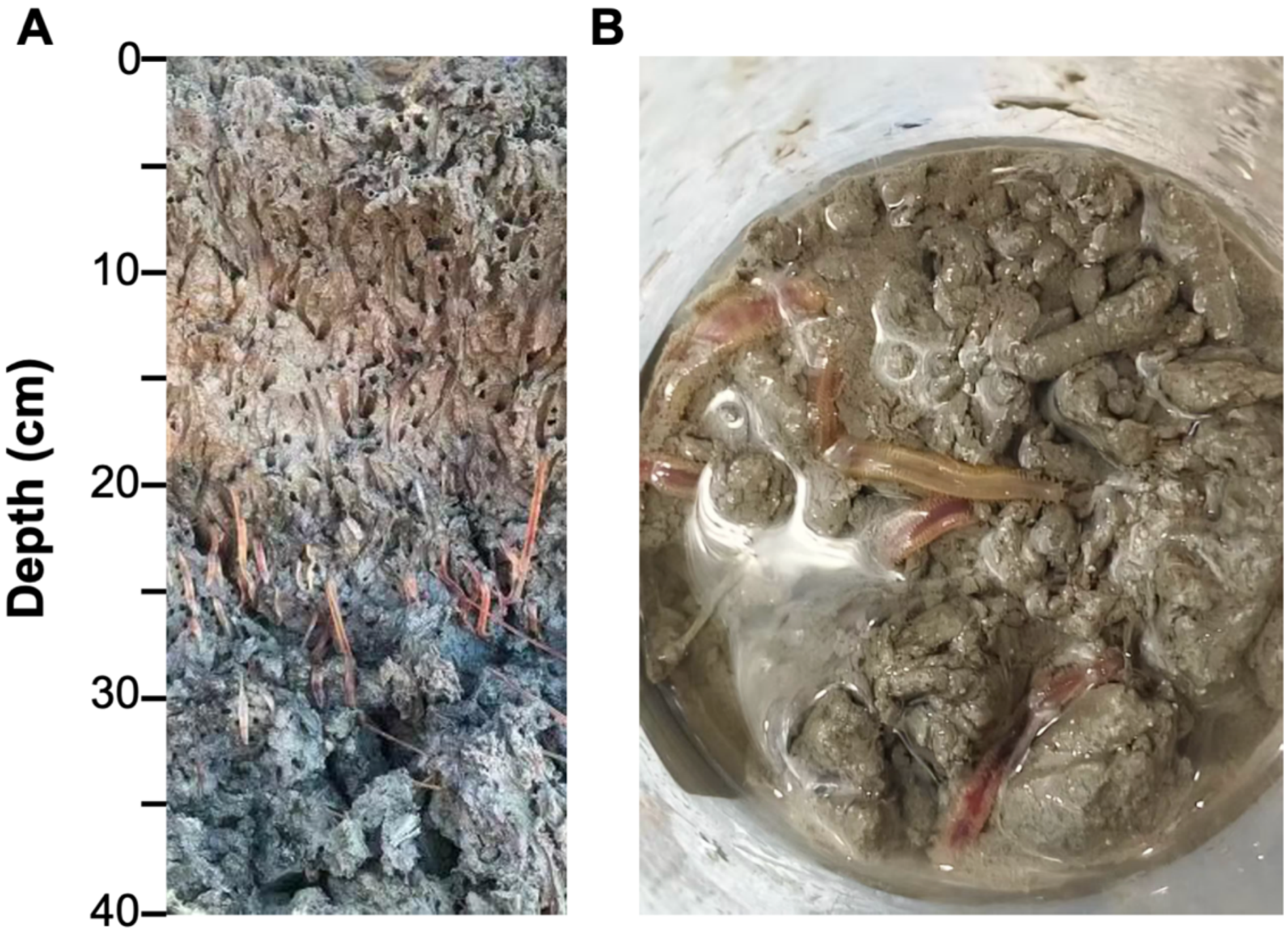

Figure S2. Photos of (A) sediment profile and (B) core-top view showing the intense bioturbation in the early-stage seep sediments. Figure A was adapted from Liang et al., 2026 (30).

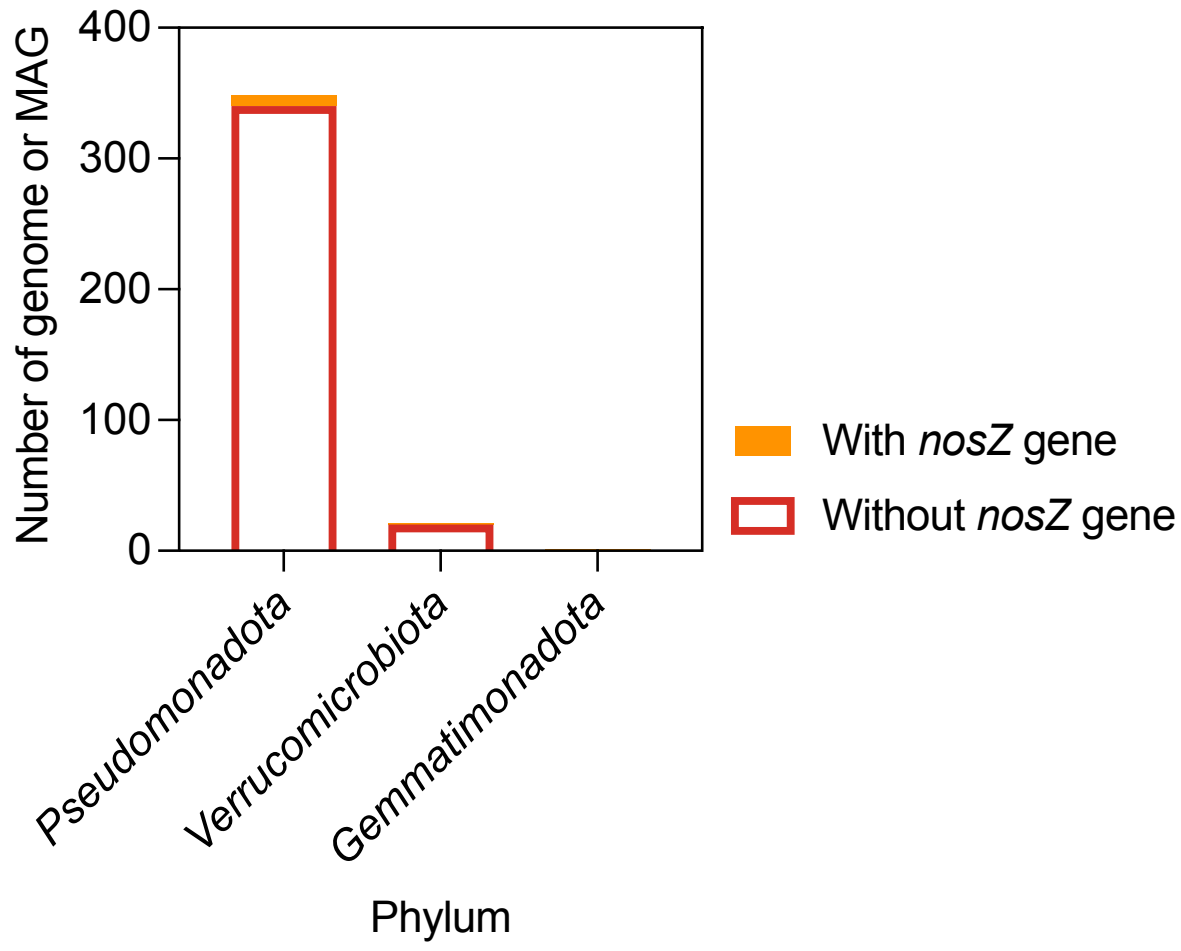

Figure S3. Numbers of methane-oxidizing bacterial (MOB) genomes or metagenome-assembled genomes (MAGs) with and without the *nosZ* gene. The phylum *Gemmatimonadota* is represented by only one genome (with *nosZ* gene) and is therefore barely visible in the figure. Data were compiled from Awala et al., (2024) (31).

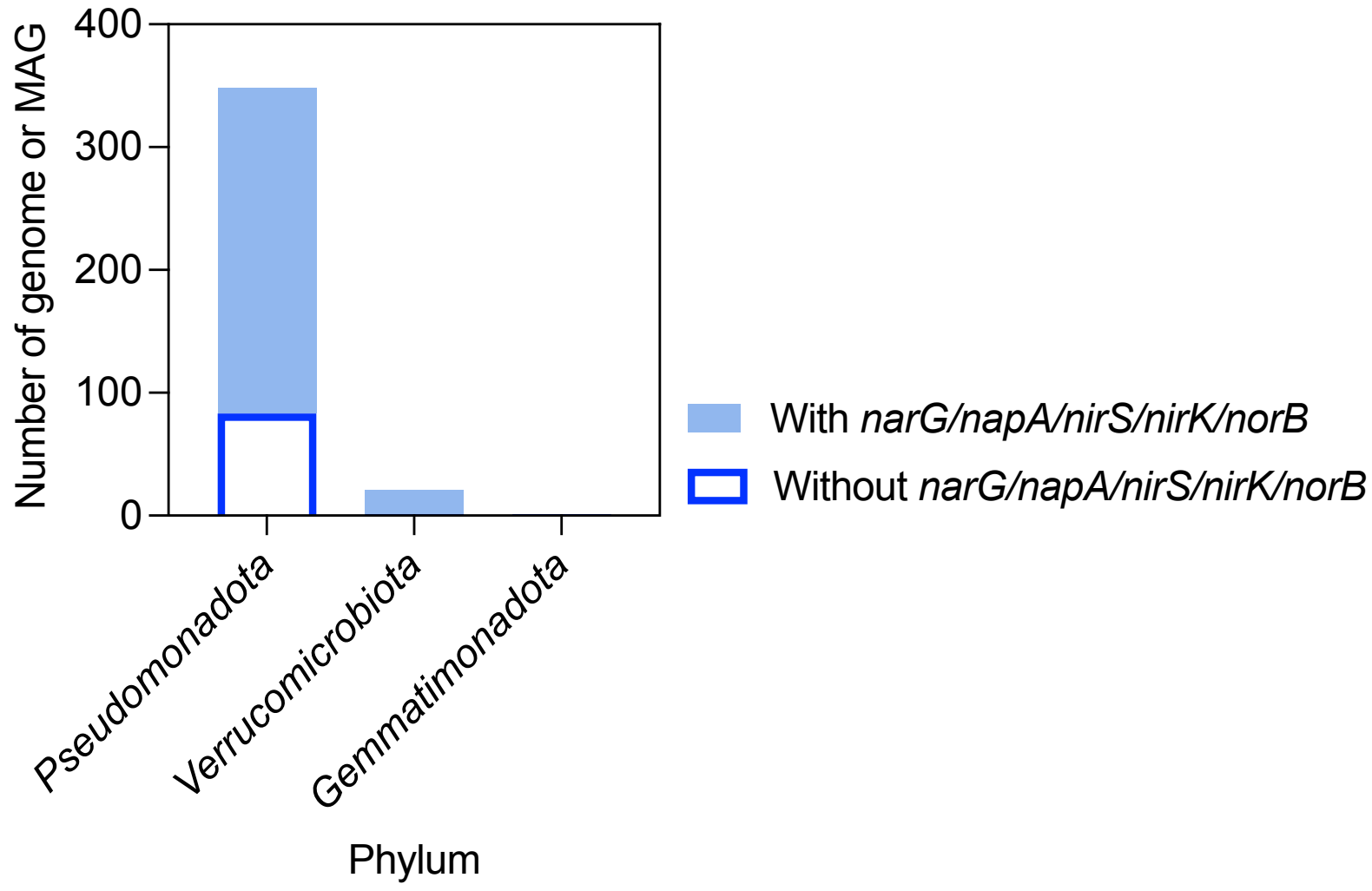

Figure S4. Numbers of methane-oxidizing bacterial (MOB) genomes or metagenome-assembled genomes (MAGs) with and without denitrification gene expect *nosZ* gene (*narG*, *napA*, *nirS*, *nirK*, *norB* gene). The phylum *Gemmatimonadota* is represented by only one genome (without *narG/napA/nirS/nirK/norB* gene) and is therefore barely visible in the figure. Data were compiled from Awala et al., (2024) (31).

Table S1. Potential nitrate/nitrite-coupled methane oxidation rate in early-stage seep and mature seep sediment

Station	Depth	Potential rate
	cm	nmol C cm^{-3} h^{-1}
Early-stage seep	0–2	1.64 ± 0.34
	2–4	7.27 ± 0.58
	4–6	0.16 ± 0.05
	6–8	0.14 ± 0.03
	8–12	7.66 ± 0.41
	12–16	10.48 ± 3.12
Mature seep	0–2	0.14 ± 0.04
	2–4	0.06 ± 0.01
	4–6	0.67 ± 0.26
	6–8	0.47 ± 0.20

Table S2. Nitric oxide reductases (NOR) class annotated with HHM model (28, 32)

MAG_ID	Target_Name	NOR Class	E_value	Score	Bias
bin1138	k255_2504943_19	bNOR	6.40E-123	408.9	48.7
bin1138	k255_2504943_19	cNOR	7.00E-167	553.6	56.9
bin1138	k255_6456518_6	cNOR	3.00E-15	53.7	7.6
bin1138	k255_7866405_2	eNOR	4.30E-69	231.3	42.4
bin1138	k255_7866405_2	eNOR_subII	2.20E-20	71.3	0.4
bin1138	k255_7866405_2	nNOR_subII	2.10E-14	51.5	0.2
bin1138	k255_7866405_3	qNOR	3.30E-70	234.8	55.1
bin1138	k255_7866405_3	qNOR	1.30E-47	160.2	7
bin1138	k255_7866405_3	sNOR	3.20E-22	76.5	48.8
bin1138	k255_7866405_3	sNOR_subII	2.00E-17	61.2	0.2
bin1266	k255_4330598_6	eNOR	5.70E-69	230.8	42.3
bin1266	k255_4872128_33	eNOR	3.70E-24	82.8	47.2
bin1266	k255_4872128_33	eNOR	4.50E-23	79.2	45.3
bin1266	k255_4872128_34	eNOR_subII	1.20E-20	72	0.5
bin1266	k255_4872128_34	gNOR	2.60E-14	50.8	38.9
bin1269	k255_1897416_3	nNOR	3.60E-14	50.2	47.3
bin1269	k255_1897416_3	nNOR_subII	1.90E-14	51.5	0.1
bin1269	k255_1897416_3	qNOR	6.20E-296	981.1	47.5
bin1269	k255_1897416_3	qNOR	3.40E-67	224.7	55.5
bin1269	k255_1897416_4	sNOR	5.10E-26	88.9	48
bin1269	k255_1897416_4	sNOR	1.30E-23	81	49.2
bin1269	k255_1897416_4	sNOR	2.60E-20	70	53.6
bin1269	k255_2054845_3	sNOR_subII	1.30E-17	61.6	0.3
bin1269	k255_2054845_3	sNOR_subII	8.90E-15	52.4	0.1
bin1269	k255_2056124_4	bNOR	4.60E-122	406	47.1
bin1269	k255_2056124_5	eNOR	3.00E-70	235.1	41
bin1269	k255_2056124_5	eNOR	2.10E-22	77.2	46.1
bin1269	k255_2056124_5	eNOR_subII	1.30E-20	72	0.8
bin1269	k255_4327785_22	nNOR_subII	1.60E-14	51.9	0.3
bin1269	k255_4327785_22	sNOR	4.00E-22	76.1	47.5
bin1269	k255_5400269_17	sNOR	4.70E-20	69.3	52.8
bin1269	k255_5400269_17	sNOR_subII	2.20E-17	61	0.3
bin1269	k255_5400269_18	cNOR	4.20E-167	553.6	57
bin1269	k255_5400269_18	qNOR	4.10E-70	233.8	54.9
bin1197	k255_3703937_104	bNOR	6.10E-21	72.1	4.5
bin1197	k255_3703937_105	eNOR	1.20E-23	81	46.2
bin1197	k255_3703937_105	eNOR	8.90E-23	78.2	45.7

bin1197	k255_643562_15	sNOR	7.70E-21	71.7	52.8
bin1197	k255_643562_15	sNOR	6.10E-20	68.7	52.1
bin1197	k255_643562_16	bNOR	8.30E-120	398.5	47.7
bin1197	k255_643562_16	cNOR	1.10E-165	549.5	56.9
bin1209	k255_4761325_14	cNOR	1.20E-66	223	55.7
bin1486	k255_3159730_7	bNOR	1.50E-116	386.9	51.6
bin1486	k255_3159730_7	cNOR	1.70E-167	554.6	59.6
bin1486	k255_3159730_7	cNOR	5.50E-18	61.8	7.9
bin1486	k255_3159730_8	eNOR	2.30E-68	227.9	46.5
bin1486	k255_3159730_8	eNOR_subII	6.40E-20	68.8	0.9
bin1486	k255_3559023_6	qNOR	4.10E-68	226.9	59
bin1486	k255_3559023_6	qNOR	3.00E-51	171.2	7.6
bin1486	k255_4420447_1	sNOR	1.90E-22	76.2	52.7
bin1486	k255_4420447_1	sNOR_subII	1.10E-16	57.7	0.8
bin631	k255_134411_1	bNOR	5.60E-114	379.4	50
bin631	k255_134411_1	cNOR	4.40E-67	224.6	54
bin631	k255_264263_33	eNOR	2.10E-59	199.4	45
bin631	k255_264263_33	eNOR	3.70E-24	83	45.1
bin631	k255_2757001_7	eNOR_subII	1.20E-20	72.2	0.1
bin631	k255_2757001_7	gNOR	2.50E-15	54.3	40.9
bin631	k255_2757001_7	nNOR_subII	5.40E-16	56.7	0.2
bin631	k255_2757001_8	qNOR	1.70E-294	976.5	46.4
bin631	k255_2757001_8	sNOR	7.40E-22	75.3	52
bin631	k255_2757001_8	sNOR	6.40E-21	72.2	52.8
bin631	k255_2757001_8	sNOR_subII	5.30E-18	63.1	0.5
bin2775	k255_6335637_10	eNOR	2.70E-23	79.6	46.7
bin2775	k255_6335637_10	eNOR_subII	8.20E-21	72.2	0.1
bin2775	k255_6335637_9	gNOR	2.20E-15	54	43.1
bin2775	k255_6335637_9	nNOR_subII	6.90E-16	55.9	0.2
bin2775	k255_6335637_9	sNOR	3.40E-21	72.6	53.4
bin2775	k255_7099345_19	sNOR	1.50E-20	70.5	54.6
bin2775	k255_7099345_19	sNOR_subII	1.40E-18	64.4	0.3
bin274	k255_7567675_8	cNOR	8.20E-67	223.4	54.5
bin274	k255_7567675_9	eNOR	3.70E-23	79.3	45.3
bin274	k255_7567675_9	gNOR	7.50E-16	55.7	43
bin274	k255_7683445_20	qNOR	9.90E-293	970.3	48.1
bin274	k255_7683445_20	sNOR	4.00E-21	72.5	53
bin274	k255_7683445_20	bNOR	2.40E-116	386.8	51
bin274	k255_7683445_21	eNOR	5.60E-61	204	45

Table S3. Distribution of denitrification genes in isolates and metagenome-assembled genomes (MAG) of *Methyloprofundus* genus. Published genomes and MAGs were compiled from Awala et al., (2024) (31).

			Methane oxidation[#]						Nitrate reduction[#]								
GenBank accession	CP (%)[##]	CT (%)[##]	*pmoA*	*pmoB*	*pmoC*	*mmoX*	*mmY*	*mmoZ*	*napA*	*napB*	*narG*	*narH*	*nirK*	*nirS*	*norB*	*norC*	*nosZ*
bin1138 (This study)	93.92	4.71	1	1	1	1	1	1	0	0	0	0	1	1	1	1	0
bin1266 (This study)	96.72	3.79	1	1	1	1	1	1	0	0	1	1	1	0	0	0	0
bin1269 (This study)	90.38	6.56	1	1	1	1	1	1	0	0	0	0	1	0	1	1	0
bin1197 (This study)	82.41	5.48	1	1	1	0	0	0	0	0	1	1	1	0	0	0	0
bin1209 (This study)	81.75	1.38	0	0	0	0	0	0	0	0	1	1	1	0	1	1	0
bin1486 (This study)	60.16	5.11	0	0	0	0	0	0	0	0	0	0	0	0	1	1	0
GCA_023146175.1	91.77	0.87	1	1	1	0	0	0	0	0	1	1	1	0	1	0	0
GCA_019428975.1	73.3	1.92	1	1	1	1	0	0	0	0	0	0	1	0	0	0	0
GCA_016592635.1	99.7	1.21	1	1	1	0	0	0	0	0	1	1	1	0	1	1	0
GCA_014859465.1	100	6.9	1	1	1	1	1	1	0	0	0	0	0	1	0	0	0
GCA_013138555.1	78.1	4.97	0	0	1	1	1	1	0	0	0	0	1	0	1	0	0

GCA_012960135.1	87.85	1.21	1	1	1	0	0	0	0	0	1	1	1	0	0	0	0
GCA_002072955.1	99.7	2.67	1	1	1	0	0	0	0	0	1	1	1	0	1	1	0
GCA_900128525.1	97.5	1.84	1	1	1	0	0	0	0	0	1	1	1	0	1	1	0
GCA_020996975.1	95.3	0.78	1	1	1	0	0	0	0	0	1	1	1	0	0	0	0
GCA_020996955.1	95	0.78	1	1	1	0	0	0	0	0	1	1	1	0	0	0	0
GCA_020996935.1	96.5	0.69	1	1	1	0	0	0	0	0	1	1	1	0	0	0	0
GCA_020996915.1	96.8	2.67	1	1	1	0	0	0	0	0	1	1	1	0	0	0	0
GCA_020996895.1	96.7	0.86	1	1	1	0	0	0	0	0	1	1	1	0	0	0	0
GCA_020996875.1	96.7	1.03	1	1	1	0	0	0	0	0	1	1	1	0	0	0	0
GCA_020996855.1	96.5	1.38	1	1	1	0	0	0	0	0	1	1	1	0	0	0	0
GCA_020996835.1	96	0.69	1	1	1	0	0	0	0	0	1	1	1	0	0	0	0
GCA_020996815.1	96.7	1.55	1	1	1	0	0	0	0	0	1	1	1	0	0	0	0
GCA_020996795.1	93.9	0.69	1	1	1	0	0	0	0	0	1	1	1	0	0	0	0
GCA_020996775.1	95.3	1.38	1	1	1	0	0	0	0	0	1	1	1	0	0	0	0
GCA_020996755.1	95.3	0.69	1	1	1	0	0	0	0	0	1	1	1	0	0	0	0
GCA_020996735.1	97	0.69	1	1	1	0	0	0	0	0	1	1	1	0	0	0	0

GCA_020996715.1	95	0.69	1	1	1	0	0	0	0	0	1	1	1	0	0	0	0
GCA_020996695.1	96.3	0.86	1	1	1	0	0	0	0	0	1	1	1	0	0	0	0
GCA_020996645.1	96.7	0.69	1	1	1	0	0	0	0	0	1	1	1	0	0	0	0
GCA_020996635.1	97	0.69	1	1	1	0	0	0	0	0	1	1	1	0	0	0	0
GCA_020996625.1	95.3	0.69	1	1	1	0	0	0	0	0	1	1	1	0	0	0	0
GCA_020996615.1	96	0.69	1	1	1	0	0	0	0	0	1	1	1	0	0	0	0
GCA_019722015.1	98.5	4.91	1	1	1	0	0	0	0	0	1	1	1	0	1	1	0
GCA_019721995.1	96	1.38	1	1	1	0	0	0	0	0	1	1	1	0	1	0	0
GCA_018697995.1	78.08	1.15	1	1	1	0	0	0	0	0	1	1	1	0	0	0	0
GCA_018672195.1	95.64	1.67	1	1	1	0	0	0	0	0	1	1	1	0	0	0	0
GCA_018669355.1	99.37	1.67	1	1	1	0	0	0	0	0	1	1	1	0	0	0	0
GCA_018668785.1	99.6	1.67	1	1	1	0	0	0	0	0	1	1	1	0	0	0	0
GCA_018667815.1	98.75	1.32	1	1	1	0	0	0	0	0	1	1	1	0	0	0	0
GCA_018661125.1	96.95	1.75	1	1	1	0	0	0	0	0	0	0	1	0	0	0	0
GCA_018654675.1	93.35	3.26	1	1	1	0	0	0	0	0	1	1	1	0	0	0	0
GCA_015659575.1	98.33	1.28	1	1	1	0	0	0	0	0	1	1	1	0	0	0	0

GCA_008030255.1	95.5	4.97	1	1	1	0	0	0	0	0	1	1	1	1	1	1	0
GCA_008030245.1	95.95	5.35	1	1	1	0	0	0	0	0	1	1	1	1	1	1	0
GCA_008030235.1	96.4	3.6	1	1	1	0	0	0	0	0	1	1	1	0	1	1	0
GCA_008030225.1	95.8	3.88	1	1	1	0	0	0	0	0	1	1	1	0	1	1	0
GCA_008030215.1	95.8	2.99	1	1	1	0	0	0	0	0	1	1	1	0	1	1	0
GCA_008030155.1	96.3	3.18	1	1	1	0	0	0	0	0	1	1	1	0	1	1	0
GCA_008030145.1	95.1	3.1	1	1	1	0	0	0	0	0	1	1	1	0	1	1	0
GCA_008030135.1	95.3	1.92	1	1	1	0	0	0	0	0	1	1	1	0	1	1	0
GCA_008030125.1	94.2	5.74	1	1	1	0	0	0	0	0	1	1	1	0	1	1	0
GCA_008030115.1	95.32	5.02	1	1	1	0	0	0	0	0	1	1	1	1	1	1	0
GCA_002189065.1	98.2	1.16	1	1	1	0	0	0	0	0	1	1	1	0	1	1	0

[#]1 indicates the presence of the gene, and 0 indicates its absence.

[##]CP: completeness; CT: contamination

Table S4. Potential N_2O production rates in the early-stage and mature seep under nitrate addition only (NO_3^-) and both nitrate and methane addition (NO_3^- + CH_4) treatments.

Station	Depth	NO_3^-	NO_3^- + CH_4
	cm	nmol N cm^{-3} h^{-1}	nmol N cm^{-3} h^{-1}
Early-stage seep	0-2	0.44 ± 0.02	0.56 ± 0.08
	2-4	1.40 ± 0.18	2.04 ± 0.18
	4-6	1.70 ± 0.21	1.96 ± 0.16
	6-8	1.15 ± 0.23	1.31 ± 0.09
	8-12	0.44 ± 0.09	0.60 ± 0.05
	12-16	0.48 ± 0.17	0.64 ± 0.06
Mature seep	0-2	n.a.	n.a.
	2-4	0.13 ± 0.04	0.08 ± 0.05
	4-6	0.44 ± 0.06	0.53 ± 0.03
	6-8	0.32 ± 0.05	0.31 ± 0.09

n.a.: not applicable

References

1. Thamdrup B, Dalsgaard T. 2002. Production of N-2 through anaerobic ammonium oxidation coupled to nitrate reduction in marine sediments. Applied and Environmental Microbiology 68:1312-1318.
2. Dalsgaard T, Canfield DE, Petersen J, Thamdrup B, Acuna-Gonzalez J. 2003. N-2 production by the anammox reaction in the anoxic water column of Golfo Dulce, Costa Rica. Nature 422:606-608.
3. Trimmer M, Nicholls JC, Deflandre B. 2003. Anaerobic ammonium oxidation measured in sediments along the Thames estuary, United Kingdom. Applied and environmental microbiology 69:6447-6454.
4. Yao X, Wang J, He M, Liu Z, Zhao Y, Li Y, Chi T, Lin Z, Zheng P, Jetten M, Hu B-L. 2024. Methane-dependent complete denitrification by a single Methylomirabilis bacterium. Nature Microbiology 9:1-13.
5. Haroon MF, Hu S, Shi Y, Imelfort M, Keller J, Hugenholtz P, Yuan Z, Tyson GW. 2013. Anaerobic oxidation of methane coupled to nitrate reduction in a novel archaeal lineage. Nature 500:567-570.
6. Ettwig KF, Butler MK, Le Paslier D, Pelletier E, Mangenot S, Kuypers MMM, Schreiber F, Dutilh BE, Zedelius J, de Beer D, Gloerich J, Wessels HJCT, van Alen T, Luesken F, Wu ML, van de Pas-Schoonen KT, Op den Camp HJM, Janssen-Megens EM, Francoijs K-J, Stunnenberg H, Weissenbach J, Jetten MSM, Strous M. 2010. Nitrite-driven anaerobic methane oxidation by oxygenic bacteria. Nature 464:543-548.
7. Bowles MW, Samarkin VA, Joye SB. 2011. Improved measurement of microbial activity in deep-sea sediments at in situ pressure and methane concentration. Limnology and Oceanography: Methods 9:499-506.
8. Zhuang G-C, Xu L, Liang Q, Fan X, Xia Z, Joye SB, Wang F. 2019. Biogeochemistry, microbial activity, and diversity in surface and subsurface deep-sea sediments of South China Sea. Limnology and Oceanography 64:2252-2270.
9. Joye SB, Boetius A, Orcutt BN, Montoya JP, Schulz HN, Erickson MJ, Lugo SK. 2004. The anaerobic oxidation of methane and sulfate reduction in sediments from Gulf of Mexico cold seeps. Chemical Geology 205:219-238.
10. Chen F, Zheng Y, Hou L, Niu Y, Gao D, An Z, Zhou J, Yin G, Dong H, Han P. 2021. Microbial abundance and activity of nitrite/nitrate-dependent anaerobic methane oxidizers in estuarine and intertidal wetlands: Heterogeneity and driving factors. Water research 190:116737.
11. Hinshaw SE, Dahlgren RA. 2013. Dissolved nitrous oxide concentrations and fluxes from the eutrophic San Joaquin River, California. Environmental science & technology 47:1313-1322.
12. Weiss RF, Price B. 1980. Nitrous oxide solubility in water and seawater. Marine chemistry 8:347-359.
13. Bolger AM, Lohse M, Usadel B. 2014. Trimmomatic: a flexible trimmer for Illumina sequence data. Bioinformatics 30:2114-20.

14. Li D, Liu CM, Luo R, Sadakane K, Lam TW. 2015. MEGAHIT: an ultra-fast single-node solution for large and complex metagenomics assembly via succinct de Bruijn graph. Bioinformatics 31:1674-6.
15. Hyatt D, Chen G-L, LoCascio PF, Land ML, Larimer FW, Hauser LJ. 2010. Prodigal: prokaryotic gene recognition and translation initiation site identification. BMC bioinformatics 11:1-11.
16. Kanehisa M, Furumichi M, Tanabe M, Sato Y, Morishima K. 2017. KEGG: new perspectives on genomes, pathways, diseases and drugs. Nucleic acids research 45:D353-D361.
17. Aramaki T, Blanc-Mathieu R, Endo H, Ohkubo K, Kanehisa M, Goto S, Ogata H. 2020. KofamKOALA: KEGG Ortholog assignment based on profile HMM and adaptive score threshold. Bioinformatics 36:2251-2252.
18. Hou J, Sievert S, Wang Y, Seewald J, Natarajan V. 2020. Microbial succession during the transition from active to inactive stages of deep-sea hydrothermal vent sulfide chimneys. Microbiome 8:102.
19. Kang DD, Li F, Kirton E, Thomas A, Egan R, An H, Wang Z. 2019. MetaBAT 2: an adaptive binning algorithm for robust and efficient genome reconstruction from metagenome assemblies. PeerJ 7:e7359.
20. Wu Y-W, Tang Y-H, Tringe SG, Simmons BA, Singer SW. 2014. MaxBin: an automated binning method to recover individual genomes from metagenomes using an expectation-maximization algorithm. Microbiome 2:1-18.
21. Alneberg J, Bjarnason BS, De Bruijn I, Schirmer M, Quick J, Ijaz UZ, Lahti L, Loman NJ, Andersson AF, Quince C. 2014. Binning metagenomic contigs by coverage and composition. Nature methods 11:1144-1146.
22. Sieber CM, Probst AJ, Sharrar A, Thomas BC, Hess M, Tringe SG, Banfield JF. 2018. Recovery of genomes from metagenomes via a dereplication, aggregation and scoring strategy. Nature microbiology 3:836-843.
23. Parks DH, Rinke C, Chuvochina M, Chaumeil P-A, Woodcroft BJ, Evans PN, Hugenholtz P, Tyson GW. 2017. Recovery of nearly 8,000 metagenome-assembled genomes substantially expands the tree of life. Nature microbiology 2:1533-1542.
24. Olm MR, Brown CT, Brooks B, Banfield JF. 2017. dRep: a tool for fast and accurate genomic comparisons that enables improved genome recovery from metagenomes through de-replication. The ISME journal 11:2864-2868.
25. Chaumeil P-A, Mussig AJ, Hugenholtz P, Parks DH. 2020. GTDB-Tk: a toolkit to classify genomes with the Genome Taxonomy Database. Oxford University Press.
26. Parks DH, Chuvochina M, Rinke C, Mussig AJ, Chaumeil P-A, Hugenholtz P. 2021. GTDB: an ongoing census of bacterial and archaeal diversity through a phylogenetically consistent, rank normalized and complete genome-based taxonomy. Nucleic Acids Research 50:D785-D794.
27. Parks DH, Imelfort M, Skennerton CT, Hugenholtz P, Tyson GW. 2015. CheckM: assessing the quality of microbial genomes recovered from isolates, single cells, and metagenomes. Genome research 25:1043-1055.

28. Murali R, Hemp J, Gennis RB. 2022. Evolution of quinol oxidation within the heme-copper oxidoreductase superfamily. Biochimica et Biophysica Acta (BBA) - Bioenergetics 1863:148907.

29. Aroney ST, Newell RJ, Nissen JN, Camargo AP, Tyson GW, Woodcroft BJ. 2025. CoverM: read alignment statistics for metagenomics. Bioinformatics 41:btaf147.

30. Liang Q, Deng L, Xie R, Liu X, Xiao X, Hou J, Wang J, Sui W, Lu N, Tong Z, Huang D, Wang Y, Han Y, Zhao J, Guo B, Zhang W, Geng M, Ren T, Ye W, Xiong Z, Dong L, Ruff SE, Meile C, Tao J, Dong X, Wang F. 2026. Rapid de novo assembly of animal-microbe biofilter to mitigate seabed methane leakage. National Science Review doi:10.1093/nsr/nwag266.

31. Awala SI, Gwak J-H, Kim Y, Jung M-Y, Dunfield PF, Wagner M, Rhee S-K. 2024. Nitrous oxide respiration in acidophilic methanotrophs. Nature Communications 15:4226.

32. Murali R, Pace LA, Sanford RA, Ward LM, Lynes MM, Hatzenpichler R, Lingappa UF, Fischer WW, Gennis RB, Hemp J. 2024. Diversity and evolution of nitric oxide reduction in bacteria and archaea. Proc Natl Acad Sci U S A 121:e2316422121.
